# Structural basis for the homotypic fusion of chlamydial inclusions by the SNARE-like protein IncA

**DOI:** 10.1038/s41467-019-10806-9

**Published:** 2019-06-21

**Authors:** Gino Cingolani, Michael McCauley, Anna Lobley, Alexander J. Bryer, Jordan Wesolowski, Deanna L. Greco, Ravi K. Lokareddy, Erik Ronzone, Juan R. Perilla, Fabienne Paumet

**Affiliations:** 1 Thomas Jefferson University, Department of Biochemistry and Molecular Biology, Philadelphia, PA 19107 USA; 2 Institute of Biomembranes and Bioenergetics, National Research Council, Via Amendola 165/A, 70126 Bari, Italy; 3 Thomas Jefferson University, Department of Microbiology and Immunology, Philadelphia, PA 19107 USA; 4 The University of Delaware, Department of Chemistry and Biochemistry, Newark, DE 19716 USA; 5 Present Address: Janssen Research and Development, Spring House, PA 19477 USA; 6 Present Address: VUE Health, Boston, MA 02110 USA

**Keywords:** X-ray crystallography, Membrane fusion, Bacterial pathogenesis, Bacterial structural biology, Cellular microbiology

## Abstract

Many intracellular bacteria, including *Chlamydia*, establish a parasitic membrane-bound organelle inside the host cell that is essential for the bacteria’s survival. *Chlamydia trachomatis* forms inclusions that are decorated with poorly characterized membrane proteins known as Incs. The prototypical Inc, called IncA, enhances *Chlamydia* pathogenicity by promoting the homotypic fusion of inclusions and shares structural and functional similarity to eukaryotic SNAREs. Here, we present the atomic structure of the cytoplasmic domain of IncA, which reveals a non-canonical four-helix bundle. Structure-based mutagenesis, molecular dynamics simulation, and functional cellular assays identify an intramolecular clamp that is essential for IncA-mediated homotypic membrane fusion during infection.

## Abstract (web summary)

*Chlamydia trachomatis* forms membrane-bound inclusions inside the host cell that are decorated with IncA, a SNARE-like protein that promotes the fusion of inclusions. Here, Cingolani et al. show that the protein folds into a non-canonical four-helix bundle and identify an intramolecular clamp required for membrane fusion.

## Introduction

The obligate intracellular pathogen *Chlamydia trachomatis* is the most frequent cause of bacterial sexually transmitted disease and infectious blindness worldwide, yet it is still considered a neglected disease pathogen by the World Health Organization^1^. *Chlamydia’s* life cycle depends on the establishment of a fast-growing parasitic organelle inside the host cell called the “inclusion”, the development of which is poorly understood. Inclusion membranes are decorated with ~60 transmembrane Inc proteins that are known to directly interact with host cell components and play a critical role in sustaining *Chlamydia*’s life cycle^2–4^. Despite their importance, Incs have remained relatively uncharacterized and little is known about their function at the molecular level. The low sequence conservation and minimal similarity that Incs share with other proteins limits the usefulness of conventional bioinformatic tools to predict their structure and function. The best-characterized chlamydial Inc is IncA. It contains two extended 3,4-hydrophobic heptad repeat segments similar to the coiled-coil regions of the eukaryotic “SNAREs” (soluble N-ethylmaleimide-sensitive factor attachment receptors), which are proteins involved in cellular transport and membrane fusion^5–9^. IncA is involved in the homotypic fusion of chlamydial inclusions^10,11^ and is the best-characterized bacterial SNARE-like protein to date.

During infection, each *Chlamydia* bacterium establishes its own inclusion inside a host cell. At high multiplicities of infection (MOI), cells contain multiple inclusions that ultimately fuse together to form one large inclusion per cell. This homotypic fusion event is important for the pathogenicity of *C. trachomatis* because natural non-fusogenic IncA mutants are replication-defective in humans and cause significantly milder disease compared with patients infected with normal fusogenic strains^12–14^. Measurements of relative chlamydial rRNA quantities in the multiplying organisms have also revealed that non-fusogenic strains grow more slowly than fusogenic strains of *Chlamydia*, which is supported by a reduced rate of protein synthesis and decreased multiplication efficiency^15^. A direct role for IncA in membrane fusion was demonstrated using microinjection of anti-IncA antibody during infection, which resulted in multiple inclusions that were unable to undergo homotypic fusion^10^. Cells infected with an IncA-deficient strain of *C. trachomatis* similarly displayed multiple inclusions at a high multiplicity of infection, further establishing that IncA is required for the homotypic fusion of inclusions^11,16^.

IncA localizes to the inclusion membrane where it can potentially interact with host and inclusion-associated proteins. Yeast two-hybrid analysis demonstrated that IncA can bind itself^10^, while immunoprecipitation also showed that IncA associates *in trans* when present on opposite membranes in a cell^5,8^. In *Chlamydia*-infected HeLa cells, the expression of ectopic IncA on the endoplasmic reticulum (ER) membrane impacts inclusion integrity and ER morphology, suggesting that IncA present on the ER interacts homotypically with IncA expressed on the inclusion and induces the fusion of both compartments^5^. While it is well-established that IncA is involved in the homotypic fusion of inclusions, the molecular mechanism of its fusogenic activity remains unknown.

Here, we describe the crystal structure of IncA, which we probed using biophysical, computational, and functional methods. We demonstrate that IncA folds into a stable, non-canonical four-helix bundle that is maintained as a monomer by intramolecular interactions. We also show that the monomeric conformation of IncA is critical for its activity during membrane fusion. Our work sheds light on a class of bacterial transmembrane proteins that control membrane fusion during infection, which is critical for *Chlamydia* pathogenicity.

## Results

### IncA folds into a non-canonical four-helix bundle

**Fig. 1:**
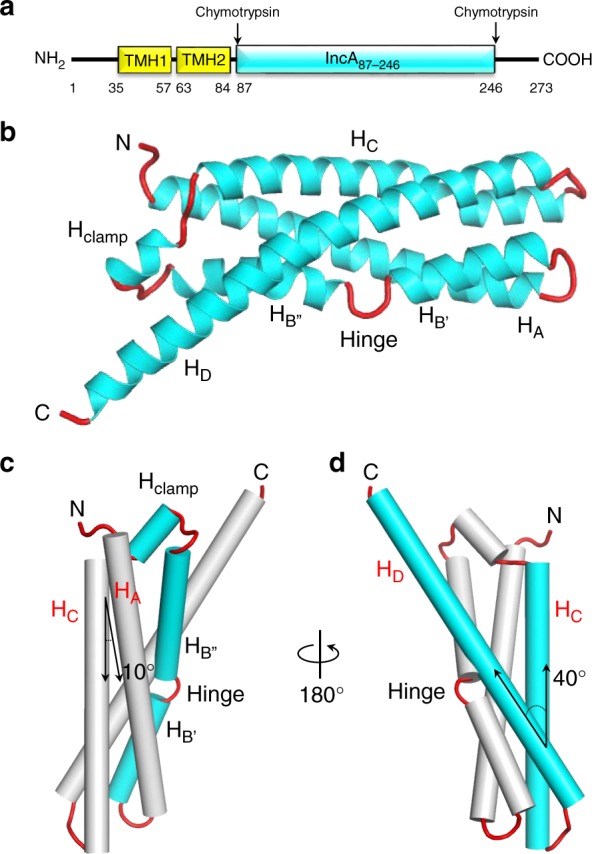
The cytosolic domain of IncA folds into a non-canonical α-helical bundle. **a** Schematic diagram of *C. trachomatis* IncA domain organization. Transmembrane helices (TMH) were predicted using the HMMTOP server^50^. The stable core encompassing residues 87–246 (IncA_87–246_) was identified by limited proteolysis of the cytosolic domain as described^8^. **b** Ribbon diagram of IncA_87–246_ with α-helices and random coiled linkers colored in cyan and red, respectively. **c**, **d** Two rotated views of IncA_87–246_, orthogonal to the representation in **b**, with α-helices shown as cylinders. **c** highlights the segmented structure of the helix H_B_, while **d** shows the relative angle between helices H_C_ and H_D_

IncA is a protein of 273 amino acids composed of a short, cytoplasmic N-terminal moiety (residues 1–34), a bilobed transmembrane domain (residues 35–84), and a long cytoplasmic C-terminal domain that ends in a tail with low complexity (residues 247–273) (Fig. 1a). The bacterial SNARE-like domains are found in the cytoplasmic C-terminal domain^5,7^. To better understand the mechanism of IncA-mediated membrane fusion, we generated high-quality crystals of a chymotryptic fragment of IncA spanning residues 87–246 (IncA_87–246_) (Supplementary Fig. 1b) and determined a crystal structure of IncA_87–246_ to an *R*_work/free_ of 14.1/16.8% at 1.12 Å resolution (Fig. 1b and Table 1). The electron density for IncA_87–246_, including the six N-terminal histidines of the affinity tag, is exceptionally clear (Supplementary Fig. 2), consistent with the low B-factor of the crystal structure (~10.8 Å^2^). We found that IncA_87–246_ adopts an asymmetric and slightly blocky conformation, somewhat similar to a four-helix bundle (Fig. 1b). Both in crystal and in solution, IncA_87–246_ exists as a monomer (Supplementary Fig. 3a, b and Supplementary Table 1), as previously observed for the full-length cytosolic domain of IncA (ΔTMD-IncA - Supplementary Fig. 1a), that sediments at equilibrium as a single species of 23.9 ± 0.8 kDa^8^. Altogether, these results suggest that the C-terminal protease-sensitive tail (res. 247–273) does not promote IncA self-association in vitro. The tertiary structure of IncA_87–246_ consists of four down-up-down-up antiparallel α-helices, named H_A_-H_D_. However, IncA_87–246_ deviates from canonical four-helix bundles in at least three aspects. First, the helix H_B_ is interrupted at position 144 by a glycine that generates two shorter helices, named H_B’_ and H_B”_ (Fig. 1b, c). We termed this break in helicity as the ‘hinge’’. Second, the loop connecting helices H_B_ and H_C_ contains a short insertion helix (res. 165–169), which we termed the ‘clamp’’ helix (H_clamp_) (Fig. 1b, c). The clamp makes numerous contacts with H_A_, H_B”_, H_C_, and H_D_ that account for a total of eight hydrogen bonds and 94 non-bonded interactions (Supplementary Fig. 4 and Supplementary Table 2). Third, while helices H_A_ and H_C_ run parallel to each other making an acute angle of ~ 10° and bonding throughout their entire length (Fig. 1c), the longest helix H_D_ makes a 40° angle from helix H_C_ pointing away from the H_clamp_ (Fig. 1d) and gives an asymmetric appearance to the helical bundle. Consistent with the unique four-helix-composition of IncA_87–246_, a search for structural relatives using DALI^17^ did not identify four-helix bundles with high structural similarities to IncA_87–246_ despite the abundance of this fold in nature. Instead, DALI found IncA_87–246_ bears structural similarity (*Z*-score = 9.0) to the talin-HIP1/R/Sla2p actin-tethering C-terminal homology (THATCH) domain core of the Huntingtin Interacting Protein 12, (PDB ID 1R0D) (Supplementary Fig. 5), which superimposes to IncA_87–246_ with a Cα root-mean-square deviation (RMSD) ~ 3.6 Å. This helical bundle, involved in the association between actin and clathrin-coated structures at the plasma membrane and trans-Golgi network^18^, also contains a clamp helix between H_B_ and H_C_, but, unlike IncA_87–246_, its C-terminal helix H_D_ is split into two α-helices, H_D_ and H_E_. Thus, the high-resolution crystal structure of the cytosolic domain of IncA reveals a non-canonical helical bundle.

**Table 1:** Crystallographic data collection, phasing, and refinement statistics

	IncA_87–246_	(IncA_87–246_)^NaI^	IncA_87–246(G144A)_
Data collection			
X-ray source	SSRL 9–2	MicroMax-007 HF	MicroMax-007 HF
Detector	Pilatus 6M PAD	Pilatus3 R 200K	Pilatus3 R 200K
Space group	P2_1_	P2_1_	P1
Cell dimensions			
* a*, *b*, *c* (Å)	38.4, 48.8, 41.7	38.4, 48.7, 41.7	41.0, 43.5, 45.8
* α*, *β*, *γ* (°)	90.0, 103.7, 90.0	90.0, 103.8, 90.0	92.9, 95.9, 93.9
Wavelength (Å)	0.98	1.54	1.54
Resolution (Å)	15–1.12 (1.16–1.12)	15–1.80 (1.83–1.80)	15–1.95 (2.02–1.95)
No. reflections (tot/unique)	875,396/ 52,606	1,788,976/ 13,917	282,344/ 20,042
* R*_sym_	5.5 (29.8)	7.6 (22.4)	4.8 (13.1)
* R*_pim_	2.3 (14.8)	1.9 (2.2)	4.8 (13.0)
Mean*I* / σ*I*	47.9 (4.7)	104.5 (23.9)	20.4 (5.8)
CC1/2	0.963	0.997	0.950
Completeness (%)	91.3 (55.0)	99.5 (99.4)	88.1 (66.3)
Redundancy	7.3 (4.5)	16.0 (12.4)	1.5 (1.5)
Wilson B-factor (Å^2^)	10.8	13.3	13.4
SAD phasing			
Number Iodine sites		16	
FOM		0.37	
Corr. of local RMS density		0.61	
Refinement			
PDB ID	6E7E		6E6A
Resolution (Å)	15–1.12		15–1.95
No. reflections	50,533		20,013
*R*_work_/*R*_free_^a^	14.1/16.8		16.9/21.2
No. of complexes in AU	1		2
No. of protein atoms	1375		2650
Ramachandran (favored/allowed/outliers)	100/0.0/0.0		99.7/0.3/0.0
R.M.S.D. from ideality			
Bond lengths (Å)	0.012		0.005
Bond angles (°)	1.649		0.860
MolProbity Score/ranking^b^	1.15/94th percentile		1.24/99th percentile
MolProbity ClashScore/ ranking^b^	3.65/5th percentile		4.71/98th percentile

Values in parentheses are for highest-resolution shells

^a^*R*_free_ was calculated using ~ 5% randomly selected reflections

^b^Percentile ranking relative to X-ray structures solved at similar resolution

### Intramolecular contacts maintain IncA as a monomer

**Fig. 2:**
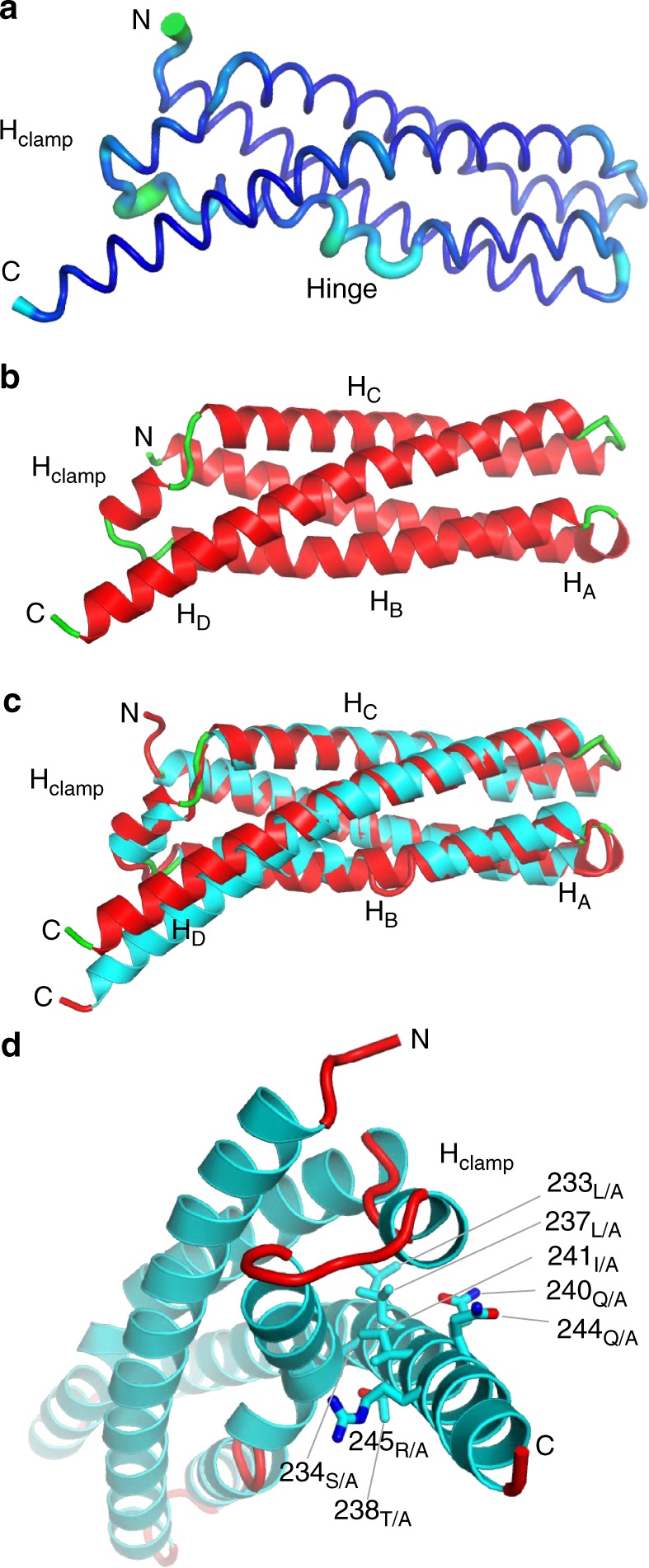
Analysis of the conformational flexibility of IncA_87–246_ reveals two features. **a** Graphic representation of IncA_87–246_ anisotropically refined B-factor plotted onto the 3D-structure: the diameter of the tube is proportional to the B-factor. The hinge region and the moiety C-terminal of the H_clamp_ (in cyan) deviate by more than two standard deviations from the mean B-factor. **b** Crystal structure of IncA_87–246(G144A)_ (protomer A), refined at 1.95 Å resolution (Table 1) with α-helices and random coiled linkers colored in red and green, respectively. **c** Secondary structure superimposition of IncA_87–246(G144A)_ (green and red) with IncA_87–246_ (red and cyan). **d** Magnified view of IncA_87–246_ viewed down the main helical axis. Residues mutated to Ala in IncA_87–246(polyA)_ are shown as sticks

To investigate how the structural determinants in IncA that deviate from a classical four-helix bundle topology affect protein flexibility and the ability to mediate homotypic fusion, we analyzed the anisotropically-refined B-factor of IncA_87–246_ (Fig. 2a). The hinge and the amino acids preceding H_clamp_ were found to have significantly higher than average B-factors (~ 26 Å^2^ vs. ~ 16 Å^2^) (Fig. 2a), possibly underscoring intrinsic flexibility. To characterize these regions, we first generated a G144A hinge mutant variant and found that IncA_87–246(G144A)_ (Supplementary Fig. 1c) remains monomeric in solution (Supplementary Fig. 3c, d and Supplementary Table 1) and it has comparable structural stability as IncA_87–246_ (Supplementary Fig. 6). IncA_87–246(G144A)_ crystallized in a triclinic space group with two IncA_87–246(G144A)_ protomers in the unit cell. Though this crystal form did not diffract as well as the previous one, we were able to collect 88.1% complete data to 1.95 Å resolution (Table 1) and determine an accurate atomic model of IncA_87–246(G144A)_ using molecular replacement. The structure, solved to a R_work/free_ of 16.9/21.2 at 1.95 Å resolution (Table 1), contains two four-helix bundles (Fig. 2b) where the interrupted α-helix H_B_ of IncA_87–246_ (Fig. 1b) is replaced by a straight continuous helix. Interestingly, the two IncA_87–246(G144A)_ protomers in the triclinic unit cell are noticeably dissimilar (RMSD 1.65 Å), with protomer B characterized by a local unfolding of helix A between residues ^100^VGSL^103^ (Supplementary Fig. 7). In both protomers, this region is not implicated in crystal contacts, which suggests IncA_87–246(G144A)_ may exist in solution in different microstates, also populated in the crystal lattice. Secondary structure superimposition of IncA_87–246_ with the reference IncA_87–246(G144A)_ protomer A reveals the G144A mutation results in significant conformational changes throughout the molecule (RMSD 1.68 Å) (Fig. 2c), especially in the helix H_D_, which is shifted upwards in IncA_87–246(G144A)_. Thus, the G144A mutation in the segmented helix B of IncA plays a global structural role in the architecture of the bundle.

To probe the second region of IncA that has a higher-than-average B-factor (Fig. 2a), we generated a mutant, IncA_87–246(polyA)_, where eight residues in H_D_ that make contact with the helices surrounding the H_clamp_ are mutated to alanine, namely L233A, S234A, L237A, T238A, Q240A, I241A, Q244A, and R245A (Fig. 2d and Supplementary Fig. 1d). Although these mutations result in a loss of 12 intramolecular bonds (Supplementary Fig. 8), IncA_87–246(polyA)_ had comparable structural stability as IncA_87–246_ (Supplementary Fig. 6). Unlike the previous two IncA constructs, IncA_87–246(polyA)_ failed to crystallize, limiting our understanding of its structure. In solution, when analyzed by AUC-SV, IncA_87–246(polyA)_ migrated as a slightly larger species than IncA_87–246_ or IncA_87–246(G144A)_, possibly consistent with a trimer or an elongated dimer (Supplementary Fig. 3e, f and Supplementary Table 1). Interestingly, IncA_87–237_, a shorter deletion construct of IncA that lacks many of the same residues on Helix D mutated in IncA_87–246(polyA)_ (Supplementary Fig. 1f), is also predominantly dimeric in solution^8^. Thus, when the intramolecular contacts generated by helix H_D_ are broken, then a higher oligomeric species spontaneously forms, suggesting that the non-canonical conformation of the H_clamp_ may function by locking the helical core of IncA in a monomeric conformation that prevents IncA from self-assembling on the inclusion membrane.

### The IncA monomer is highly stable

**Fig. 3:**
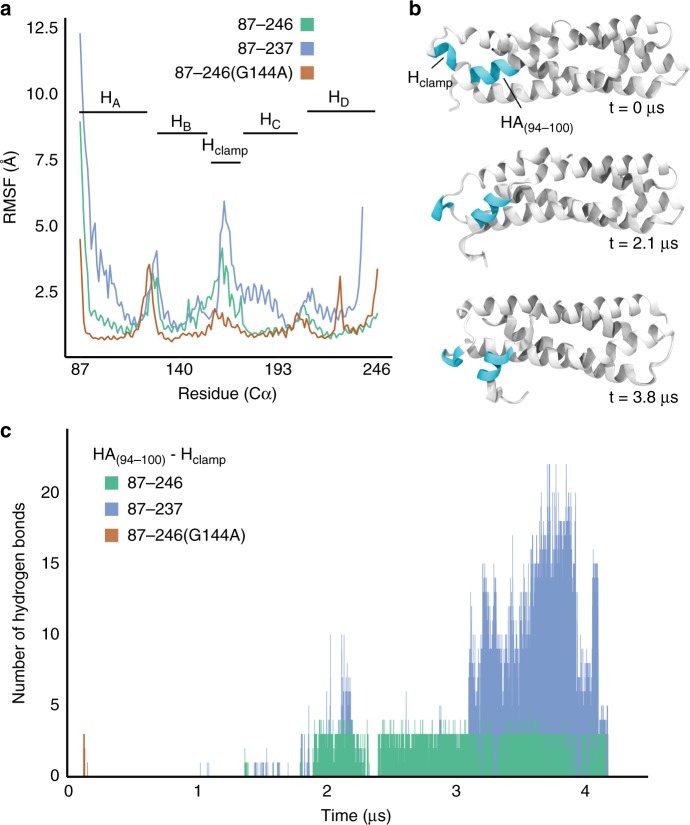
Molecular dynamics simulations show that IncA_87–246_ monomer is stable. **a** Root-mean-square fluctuation (RMSF) analysis of the three simulations performed: IncA_87–246_ (green line), IncA_87–237_ (blue line), and IncA_87–246(G144A)_ (orange line). Prior to calculating RMSF, each of the three trajectories was aligned by minimizing the root-mean-square deviation (RMSD) of backbone atoms between the equilibrated structure and every subsequent frame. **b** Three snapshots (*t* = 0 µs, *t* = 2.1 µs, *t* = 3.8 µs) of the observed conformational change during the IncA_87–237_ simulation. The two regions of interest, H_clamp_ and H_A(94–100)_, are colored in cyan. **c** Bar plot of the number of hydrogen bonds occurring between the H_clamp_ and H_A(94–100)_ over the course of all three simulations. Hydrogen bonds were identified using a contact-analysis between hydrogen, oxygen, and nitrogen atoms within a cutoff of 3.2 Å

To determine if the crystallographic structure of IncA_87–246_ represents a metastable intermediate, we subjected the 1.12 Å crystallographic model of IncA_87–246_ to equilibrium molecular dynamics simulations, along with models of IncA_87–237_ and IncA_87–246(G144A)_ (Supplementary Fig. 1). These three systems were subjected to 4 µs of equilibrium sampling to explore conformational flexibility and stability of IncA, and to probe the effects of the hinge, H_clamp_, and the C-terminus of helix H_D_ on dynamics and structure. Our simulations showed that the structure of IncA_87–246_ is highly stable and is unlikely to be a metastable intermediate. This conclusion is supported by the rigid, unchanging conformation of IncA_87–246_ throughout the equilibrium sampling at physiological conditions (310 K, 150mM NaCl) and over the entirety of the simulation (Supplementary movie 1). In contrast, IncA_87–237_, which lacks nine additional C-terminal residues, underwent a structure-wide increase in root-mean-square fluctuation (RMSF) compared to both IncA_87–246_ and IncA_87–246(G144A)_ (Fig. 3a), and experienced an eventual conformational change after ~3.2 µs of sampling (Fig. 3b and Supplementary movie 2). This conformation change involved a repositioning of H_clamp_ away from helix H_D_, toward helix H_A_, where it ultimately formed H-bonds with residues 94 to 100 of H_A_, hereinafter referred to as H_A(94–100)_. Hydrogen bonds were identified using a contact-analysis with a cutoff at 3.2 Å, between hydrogen, oxygen, and nitrogen atoms of the H_clamp_ (res. 165–169) and H_A(94–100)_. This hydrogen bond analysis was conducted for all three systems (Fig. 3c). Hydrogen bonds in IncA_87–237_ appeared transiently around 2 µs following positional fluctuations of H_A(94–100)_, and they persisted once H_A(94–100)_ repositioned permanently at 3.2 µs. Beyond the notable structural instability compared to IncA_87–246_, this result implies that the C-terminus of helix H_D_ (res. 237–246) plays a significant role in regulating the conformation of IncA_87–246_. The RMSF analysis shows that the H_clamp_ is highly flexible in all three structures (Fig. 3a). Moreover, the relatively higher magnitude of RMSF in the H_clamp_ region of IncA_87–237_ compared with that of IncA_87–246_ and IncA_87–246(G144A)_ again makes the structural effect of the C-terminus of helix H_D_ apparent.

Structural changes of IncA_87–246(G144A)_ during simulation are minimal compared to simulations of IncA_87–246_ (Supplementary movies 3 and 4). The latter observation supports the hypothesis that helix H_D_ is a key determinant of structural stability. Furthermore, noting both the lower RMSF of IncA_87–246(G144A)_ (Fig. 3a) and the dissimilarity in the position of H_D_ among IncA_87–246(G144A)_ and IncA_87–246_ (Fig. 2c), it is evident that the orientation of helix H_D_ with respect to H_clamp_ is coupled to the overall stability of each construct. Closer proximity between H_clamp_ and H_D_ in IncA_87–246(G144A)_ prevents the H_clamp_ from forming hydrogen bonds with H_A(94–100)_ resulting in a more stable conformation (Fig. 3c). The H_clamp_ of IncA_87–246_, with a significantly higher RMSF than that of IncA_87–246(G144A)_, forms hydrogen bonds with H_A(94–100)_ after 2 μs of sampling. While less-numerous and denoting no clear structural significance compared with those of IncA_87–237_, the lack of hydrogen-bonding in IncA_87–246(G144A)_ implies that the position of the C-terminus of H_D_ relative to the H_clamp_ directly modulates the behavior of H_clamp_. This finding suggests that the overall flexibility of the H_clamp_, potentially its ability to recognize regulatory proteins, is sensitive to the network of hydrogen bonds it can form with adjacent regions of H_D_ and H_A_.

Markov State models (MSMs) provide information regarding the thermodynamic stability of a protein and are typically used to characterize long timescale dynamic modes^19^, such as folding coordinates. For the trajectories in the present study (4.18 µs for IncA_87–246_ and 5.80 µs for IncA_87–237_), the high temporal resolution of the input datasets (40,000 states with each representing an interval of 100 ps), coupled with an appropriate lag time of 10 ns, resulted in four- and five-state models for IncA_87–237_ and IncA_87–246_, respectively. Models were scored and ranked for IncA_87–237_ and IncA_87–246_ separately, according to the generalized mixed Rayleigh quotient (GMRQ); the latter, a scalar value measuring the fit quality of a given MSM’s learned dynamical eigenvectors against its input data (training score) or new data (testing score)^20^. Importantly, the resulting Markov states (shown in Supplementary Fig. 9a) were different for IncA_87–237_ and IncA_87–246_. In particular, the resulting MSM of IncA_87–246_ describes just four states that are structurally similar beyond fluctuation at the termini and H_clamp_, in contrast to the five models observed for IncA_87–237_, which demonstrate the construct’s flexible conformation observed during simulation. For both of the top-scoring models, the longest implied relaxation time was <200 ns (Supplementary Fig. 9a), asserting that each of the identified Markov state interconverts on relatively short timescales. The free energy landscape (Supplementary Fig. 9b) comparing the two MSMs shows the RMSD of each state mapped to its computed free energy (∆*G*), illustrating that the sampled states of IncA_87–246_ belong to a single free energy basin. This information, coupled with the high structural similarity of each full-length Markov State and associated relaxation times, draws us to conclude that the structure of IncA_87–246_ represents a thermodynamic minimum (Supplementary Fig. 9c).

Simulation of IncA_87–246(G144A)_ revealed a spike in RMSF at residue 231 (Fig. 3a), a feature which is absent in both the IncA_87–246_ and IncA_87–237_ RMSF profiles. This result agrees with the structural plasticity of IncA_87–246(G144A)_ protomers seen in the triclinic crystal form (Table 1), which deviate significantly at the C-terminus of helix H_D_ (Fig. 2c). Interestingly, the relative spike in RMSF of the H_clamp_ region is substantially lower in IncA_87–246(G144A)_ than the other two structures. The repositioning of H_D_ that results from the G144A mutation (Fig. 2c) and its subsequent effect of lowering the flexibility of the H_clamp_ could possibly explain why IncA_87–246(G144A)_ was unable to oligomerize in solution during experiments. Altogether, these results indicate that the structure of IncA_87–246_ is highly stable and is not likely to spontaneously unfold to engage in homotypic fusion. Evidence for the importance of the C-terminal helix H_D_ in regulating IncA_87–246_ stability is illustrated through the simulation of the shorter IncA_87–237_, which underwent a conformational change, and subsequent comparative analysis of the trajectory against IncA_87–246_ and IncA_87–246(G144A)_. For all three structures, the H_clamp_ is a region of high flexibility, suggesting that it may play a role in the function of IncA. However, when G144 of the hinge is mutated to alanine, the H_D_–H_clamp_ distance narrows and results in a significantly less flexible H_clamp_ during simulation compared with IncA_87–246_ and IncA_87–237_, which may explain experimental results regarding the lack of IncA_87–246(G144A)_ oligomerization in solution (Supplementary Fig. 3 and Supplementary Table 1).

### Intramolecular contacts are critical for IncA function

**Fig. 4:**
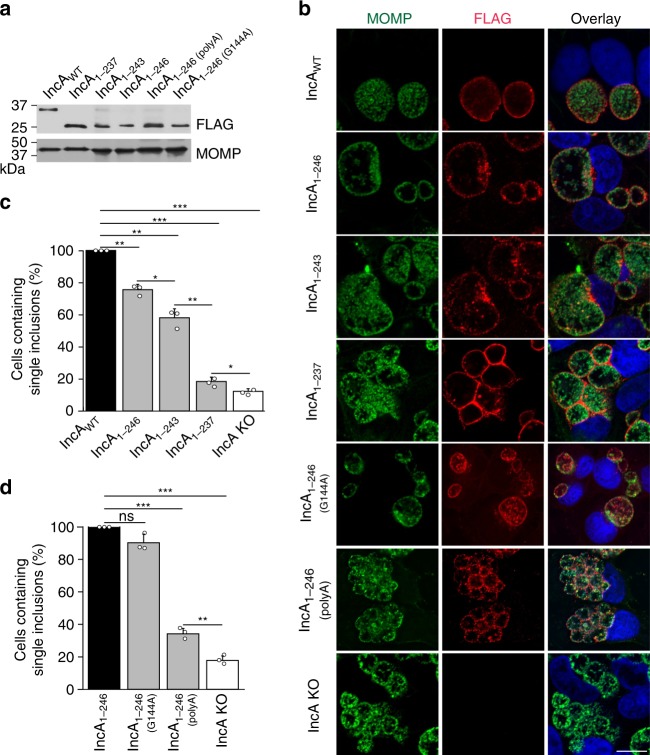
The intramolecular interactions in IncA_87–246_ are critical for its fusogenic activity. **a** IncA_mutant_-FLAG complemented-IncA KO strains express comparable amounts of IncA_mutant_-FLAG protein (Supplementary Fig. 10). HeLa cells were infected with the indicated strains for 24 h and then lysed in sample buffer. Samples were analyzed by western blot using anti-FLAG and anti-MOMP primary antibodies. *C. trachomatis* MOMP served as a loading control for infection. **b** Immunofluorescence microscopy analysis of HeLa cells infected with the indicated IncA_mutant_-FLAG complemented IncA KO strain at 24 hpi. Bacteria were labeled with anti-MOMP (green) antibody and the expression of IncA_mutant_-FLAG on the inclusion was revealed with anti-FLAG (red) antibody. DNA was stained with Hoechst (blue). The ring-like FLAG staining (red) shows that the IncA_mutant_-FLAG constructs are secreted onto the inclusion surface, indicating that any loss of fusogenic function is not due to their mislocalization. Scale bar = 10 µm. Images are representative of three independent experiments. **c** and **d** Quantification of homotypic fusion with the indicated IncA_mutant_-FLAG complemented IncA KO strain. Data were normalized to IncA_WT_-FLAG (**c**) or IncA_1–246_-FLAG (**d**) *Chlamydia*. IncA KO *Chlamydia* served as a negative control. Graphs display the means of three independent experiments ± the standard deviation. Asterisks indicate statistical significance where, * denotes *p*-values < 0.05, **denotes *p*-values < 0.01, and *** denotes *p*-values < 0.001 (two-tailed student *t*-test). Source data are provided as a Source Data file

To probe the functional importance of IncA oligomerization, we assessed the fusogenic activities of the multiple IncA constructs (IncA_87–246_, IncA_87–243_, IncA_87–237_, IncA_87–246(G144A)_, and IncA_87–246(polyA)_, Supplementary Fig. 1) during *Chlamydia* infection. We complemented an IncA knock-out (KO) *Chlamydia* strain^11^ with the different IncA mutants. All IncA mutants expressed the intact N-terminal transmembrane domain (residues 1–86) for efficient secretion to the inclusion membrane, as well as a C-terminal FLAG-tag for rapid identification (see Supplementary Table 3). Expression levels for each IncA_mutant_-FLAG protein were comparable as validated by western blot analysis (Fig. 4a and Supplementary Fig. 10). HeLa cells were infected with these strains at an MOI of 5, and the homotypic fusion of inclusions was quantified 24 h post-infection (hpi). IncA KO *Chlamydia* complemented with IncA_WT_ undergoes comparable levels of homotypic fusion as wild-type *Chlamydia* (Supplementary Fig. 11) and was used as a positive control, while the IncA KO strain was used as negative control. As shown in Fig. 4b, c, cells infected with *Chlamydia* expressing IncA_1–246_, which lacks 27 C-terminal residues (Supplementary Fig. 1), had 23% fewer cells containing single, fused inclusions compared with IncA_WT_. While this decrease is statistically significant, it demonstrates that the protease-resistant fragment IncA_1–246_ remains largely fusion-competent. When the C-terminus was further shortened, the fusogenicity of IncA continued to drop with the deletion of only three additional residues (IncA_1–243_) resulting in a drastic 43% inhibition of fusion. When six additional residues were deleted (IncA_1–237_), we observed ~82% inhibition of fusion (Fig. 4c), demonstrating a nearly complete loss of fusogenicity. Since IncA_87–237_ is mostly dimeric in solution^8^, this finding suggests that the self-assembly of IncA could impact its fusogenic activity. Altogether, these data reveal that progressive deletion of the C-terminal residues of helix H_D_ in IncA results in an increased loss of fusogenicity, which correlates with a shift towards self-assembly and a loss of monomeric IncA in solution.

To further address the potential impact of IncA oligomerization on homotypic fusion, we complemented the IncA KO *Chlamydia* strain with IncA_1–246(polyA)_, in which the residue-forming contacts on helix H_D_ have been mutagenized. We observed that IncA_1–246(polyA)_ forms oligomers in solution (Supplementary Fig. 3e, f and Supplementary Table 1). We also observed that IncA_1–246(polyA)_ was unable to drive homotypic fusion efficiently, and only ~ 33% of infected cells contained single inclusions (Fig. 4b, d). This loss of function was specific for these mutations. Cells infected with *Chlamydia* expressing another mutated IncA, IncA_1–246(G144A)_, in which the hinge was genetically eliminated, were fully fusogenic and mostly displayed a single inclusion (Fig. 4b, d). Glycine 144 is not conserved amongst the fusogenic IncA proteins, further supporting our functional data, which indicate this particular amino-acid does not play a critical role in fusion.

Altogether, these data suggest that the intramolecular contacts generated by helix H_D_ likely maintain IncA in a monomeric fusion-competent state. We observed that the IncA mutants that form oligomers in solution are all non-fusogenic when expressed on the inclusion membrane. Although the oligomerization state of IncA on the membrane is unknown, these results suggest that IncA self-association may lead to its inactivation.

### Wild-type IncA rescues fusion of non-fusogenic IncA

**Fig. 5:**
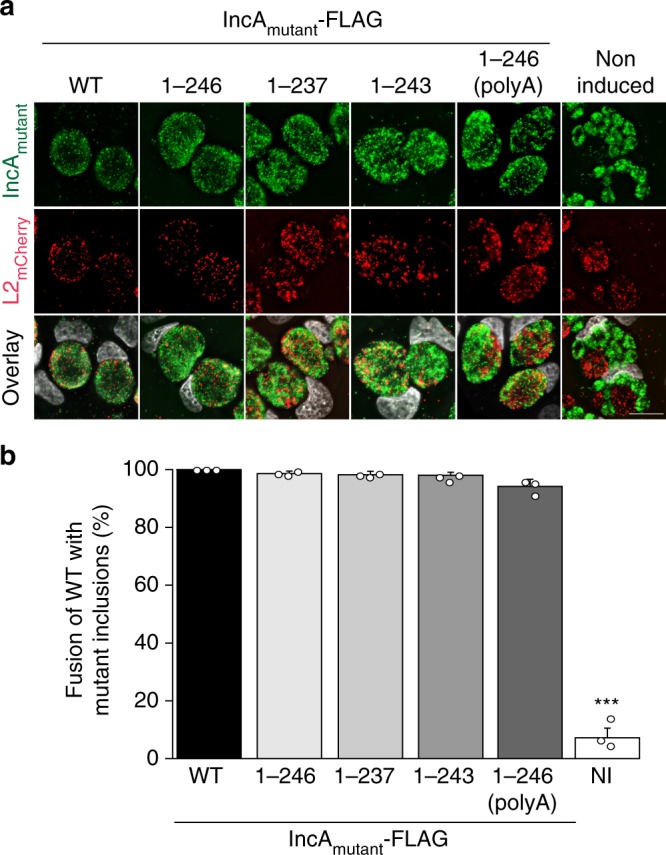
Wild-type IncA rescues the activity of non-fusogenic IncA when expressed in *trans*. **a** HeLa cells were co-infected with the indicated IncA_mutant_-FLAG complemented IncA KO strains (IncA mutant, green) at a MOI of 5 and wild-type *C. trachomatis* expressing mCherry (L2_mCherry_, red) at a MOI of 2. IncA_mutant_-FLAG expression was induced with anhydrotetracycline at 5 hpi. Not induced (DMSO) served as a negative IncA KO control. Cells were fixed at 24 hpi and DNA was stained with Hoechst (gray). L2_mCherry_ (red) and IncA_mutant_ (green) inclusions that have undergone fusion contain both red and green *Chlamydia*. Scale bar = 10 µm. **b** Quantification of heterotypic fusion between L2_mCherry_ and IncA_mutant_ inclusions. Data were normalized to IncA_WT_-FLAG. Graphs display the means of three independent experiments ± the standard deviation. Asterisks (***) denote a *p*-value < 0.001 (two-tailed student *t*-test). Source data are provided as a Source Data file

To further probe the mechanism controlling IncA function and assess the importance of *trans*-interactions between IncA present on opposing membranes, we conducted a series of co-infection experiments in which cells were infected with both non-fusogenic IncA_mutant_ complemented-IncA KO *C. trachomatis* expressing GFP (MOI 5) and wild-type *C. trachomatis* expressing mCherry (L2_mCherry_, MOI 2) (Fig. 5). Wild-type *C. trachomatis* transformed with mCherry (L2_mCherry_) undergoes the same level of fusion as wild-type *Chlamydia* (Supplementary Fig. 11). We used the IncA KO *Chlamydia* strain complemented with IncA_1–246_, and the IncA KO (non-induced) strain as positive and negative controls, respectively. As expected, IncA_1–246_ was able to promote fusion with IncA_WT_, resulting in a mixed inclusion containing both GFP and mCherry *Chlamydia* (Fig. 5a). Strikingly, the mutants for which homotypic fusion was significantly impaired (IncA_1–237_, IncA_1–243_, and IncA_1–246(polyA)_), were all able to promote “heterotypic” fusion when IncA_WT_ was present on the opposing membrane, thus resulting in complete rescue with almost all of the cells (97%, 96%, 94%, respectively) displaying single red wild-type inclusions that fused with green mutant inclusions (Fig. 5), compared with far fewer of the cells (~ 18%, 55%, and 35%, respectively) having homotypically fused inclusions (Fig. 4). These results suggest that IncA_WT_ was able to form *trans*-interactions, likely by disassembling *cis*-oligomers (see Discussion). Inclusions generated by the IncA KO *Chlamydia* strain were unable to undergo fusion even with WT inclusions (Fig. 5a, b, non-induced). This observation confirms that IncA needs to be present on both inclusion membranes to promote fusion. Altogether, these data suggest a model whereby IncA_WT_ is able to disassemble *cis* IncA_mutant_ oligomers to promote IncA_WT_**:**IncA_mutant_ fusion.

## Discussion

Fusogenic viral proteins^21^ and eukaryotic SNAREs^22–24^ have been extensively studied, and much is known about the key structural determinants and, in some cases, mechanisms of membrane fusion. A common denominator of fusogenic proteins is their intrinsic structural plasticity and ability to undergo dramatic conformational changes upon fusion, typically adopting a metastable conformation in the pre-fusogenic state and a thermodynamically stable structure post-fusion^25,26^. In contrast, the key players and mechanisms that mediate fusion of chlamydial inclusions remain largely unknown. Chlamydial inclusions are extraordinarily challenging to study due to the fragility of the lipid membrane, the poor conservation of Inc proteins, and the lack of an in vitro fusion assay. In addition, the homotypic fusion of chlamydial inclusions presents a formidable topological challenge because the proteins responsible for this event are identical on both membranes^27^. In this paper, we describe the molecular architecture of the prototypical bacterial SNARE-like protein IncA, which mediates the homotypic fusion of chlamydial inclusions^10,11^.

Our work sheds light on three aspects that are important for  deciphering the mechanisms of chlamydial inclusion membrane fusion. *First*, the cytosolic domain of IncA folds into a non-canonical four-helix bundle characterized by a segmented helix H_B_, a clamp, and a long C-terminal helix H_D_. This structure differs profoundly from the four-helix bundle structure of eukaryotic SNAREs previously used to describe IncA topology (Supplementary Fig. 12) but shares similarity with the THATCH domain core of the Huntingtin Interacting Protein 12 that mediates associations between actin and clathrin-coated structures.

Combining biochemical, biophysical, and functional methodologies, we provide evidence that the C-terminal helix H_D_ of IncA makes key intramolecular contacts with H_clamp_ and helix H_B_, thus locking the IncA bundle into a stable monomeric pre-fusion conformation. These contacts, in particular, S234, Q240, I241, and Q245, are conserved in all known fusogenic IncA proteins (i.e., *Ctr*IncA expressed by *C. trachomatis*, *Cmu*IncA expressed by *C. muridarum*, and *Cs*IncA expressed by *C. suis*), supporting the idea that this region is important for IncA function. *Second*, using molecular dynamics simulations, we establish that the crystallographic structure of the IncA cytosolic core is thermodynamically stable and unlikely to undergo major tertiary structural conformational changes spontaneously. Local conformational changes in IncA occur during membrane fusion, but they likely remain confined to regions of the bundle characterized by higher RMSD, such as the H_clamp_ and the C-terminal helix H_D_. *Third*, by assessing the fusion of the inclusions in HeLa cells infected with genetically manipulated *Chlamydia* strains, we establish a direct correlation between the monomeric state of IncA in solution and its fusogenic activity. Deletion of just nine amino acids of the C-terminal helix H_D_ results in the formation of stable IncA dimers in vitro and the drastic loss of fusogenic activity during infection. This inhibition of fusion was nonetheless completely rescued when cells infected by *Chlamydia*-expressing IncA_mutants_ were co-infected with *Chlamydia*-expressing IncA_WT_, suggesting that the full-length bundle exerts a dominant function in *trans* to restore the fusogenic activity of IncA_mutants_.

**Fig. 6:**
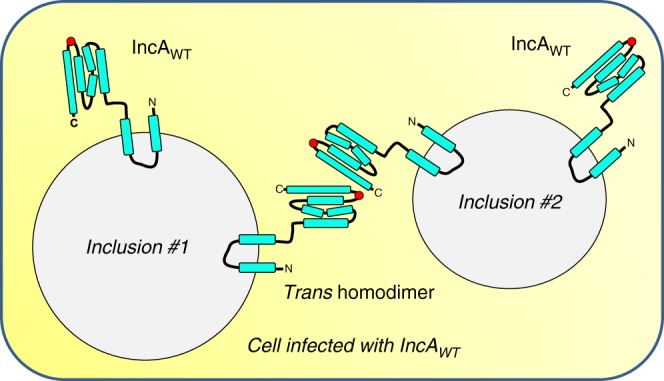
Model of IncA-mediated homotypic fusion of *C. trachomatis* inclusions. Schematic diagram of a cell infected by *C. trachomatis* that expresses IncA_WT_. Each inclusion displays monomeric IncA_WT_. The fusion of inclusion membranes requires the formation of *trans*-homodimers of IncA_WT_, which interact via the C-terminal helix H_D_ and the H_clamp_ (highlighted with a red circle)

Using these data, we postulate a model in which IncA_WT_ promotes the fusion of inclusion membranes by engaging in homotypic interactions in *trans* (i.e., *trans* homodimer in Fig. 6) mediated by the C-terminal helix H_D_. Such an interaction is unlikely to be a simple swap of the C-terminal helix because molecular dynamic simulations argue against a global unfolding of the IncA four-helix bundle. Instead, this interaction appears limited to a molecular contact between two IncA bundles. The ability of IncA_WT_ to rescue IncA_mutants_ in *trans* suggests that the association between IncAs is not restricted to the C-terminal helix and may use determinants in the H_clamp_.

The non-canonical helical structure of IncA is key to solving the topological conundrum of homotypic fusion. In this context, the intramolecular contacts within IncA C-terminal helix H_D_ and H_clamp_ are essential for its fusogenic activity as it allows for homotypic interactions to occur only in *trans*. By removing the intramolecular interactions through point mutations (IncA_1–246(polyA)_) or truncation (IncA_1–237_), we show that IncA_mutants_ self-assemble in solution, likely leading to the formation of *cis* homodimers on the inclusion membrane (Supplementary Fig. 13). Consequently, the *cis* homodimers cannot interact with IncA in *trans* and thus are unable to promote homotypic fusion.

This model is supported by a previous analysis of the oligomeric state of IncA during infection, which found that IncA can naturally switch between monomeric and oligomeric states^5,8,10^, but we cannot rule out the existence of an additional host or *Chlamydia* factor that assists in the fusogenic bundling of IncA. One possibility is that a soluble or membrane-bound regulatory protein could catalyze the formation of the IncA *trans*-complex, leading to membrane fusion. It is also possible that the unstructured moiety at the C-terminus of IncA helix H_D_ or perhaps an intramolecular interaction between the helical bundle and the N-terminal membrane-embedded hairpin stabilizes the auto-inhibitory state that keeps IncA monomeric and competent for fusion.

In summary, the structure of the cytoplasmic domain of IncA described in this study sheds light on how this unique bacterial protein engages in functional complexes to control membrane fusion. Our studies also provide an invaluable template for homology modeling of Inc-orthologs from other fusogenic and non-fusogenic *Chlamydia* strains^10,28^, and will help guide us towards a more mechanistic understanding of how mutations and sequence insertions in IncA affect its fusogenic activity.

## Methods

### Gene cloning and mutagenesis

All primers and plasmids used in this study are detailed in Supplementary Tables 4 and 5, respectively. IncA_87–246_-His_6x_ (FD578) was constructed by PCR amplification of the region Thr87 to Lys246 using primers FO515/FO516 and full-length IncA as a template. The PCR product was digested with *Nco*I and *Xho*I and ligated into pET28a. IncA_87–246(G144A)_-His_6x_ (FD915) was made using the QuikChange mutagenesis kit (Agilent) and the primers FO1067/FO1068 following the manufacturer instructions. IncA_87–246(polyA)_-His_6x_ (FD985) was constructed by PCR amplification of the region spanning Thr87 to Lys246 using primers FO1116/FO1185 and a synthetic gene containing the alanine mutations (GenScript) as the template. The PCR product was digested with *Nco*I and *Xho*I and ligated into pET28a. IncA_1–246_-FLAG (FD930) was constructed by PCR amplification of the region spanning Met1 to Lys246 using primers FO1023/FO1024 and full-length IncA as the template. The PCR product was digested with *Not*I and *Sal*I and ligated into pBOMB3-Tet. The pBOMB3-Tet plasmid (FD929) was constructed by removing the tetracycline promoter from pBOMB4-Tet with *Xho*I and *Not*I followed by ligation into pBOMB3 (FD923)^29^. IncA_1–243_-FLAG (FD944) was constructed by PCR amplification of the region spanning Met1 to Leu243 using primers FO1023/FO1143 and full-length IncA as template. The PCR product was digested with *Not*I and *Sal*I and ligated into pBOMB3-Tet. IncA_1–246(G144A)_-FLAG (FD945) was constructed by PCR amplification of the region spanning Met1 to Lys246 using primers FO1023/FO1024, and full-length IncA served as the template. The PCR product was digested with *Not*I and *Sal*I and ligated into pBOMB3-Tet. Next, the G144A mutation was introduced using the QuikChange mutagenesis kit (Agilent) and the primers FO1067/FO1068 following the manufacturer instructions. IncA_1–246(polyA)_-FLAG (FD948) was constructed by PCR amplification of the region spanning Met1 to Lys246 using primers FO818/FO1184 and a synthetic gene containing the alanine mutations (GenScript) as template. The PCR product was digested with *Not*I and *Sal*I and ligated into pBOMB3-Tet.

### Recombinant protein expression and purification

IncA_87–246_-His_6x_ (FD578), IncA_87–243_-His_6x_ (FD933), IncA_87–246(G144A)_-His_6x_ (FD915), and IncA_87–246(polyA)_-His_6x_ (FD985) were transformed into BL21(DE3) *E. coli* (Invitrogen, cat #44–0049) and grown in Luria-Bertani media to an optical density (at 600 nm) of 0.8. Protein expression was induced with 0.2 mM isopropyl β-d-thiogalactopyranoside at 16 ˚C for 20 h. After centrifugation, bacteria pellets containing IncA_87–246_-His_6x_ were resuspended in 25 mM Hepes pH 7.4, 75 mM NaCl, 10% (w/v) glycerol, 0.5 mM Tris-(2-carboxyethyl)phosphine hydrochloride (TCEP-HCl), 1 mM phenylmethylsulfonul fluoride (PMSF), 10 µM Leupeptin, and 1.5 µM Pepstatin A; IncA_87–243_-His_6x_ was resuspended in 8.45 mM Potassium phosphate mono-basic/40.5 mM Sodium phosphate di-basic pH 7.4, 200 mM NaCl, 10% (w/v) glycerol, 0.5 mM TCEP-HCl, 1 mM PMSF, 10 µM Leupeptin, and 1.5 µM Pepstatin A; IncA_87–246(G144A)_-His_6x_ was resuspended in 25 mM Hepes pH 7.4, 200 mM NaCl, 10% (w/v) glycerol, 0.5 mM TCEP-HCl, 1 mM PMSF, 10 µM Leupeptin, and 1.5 µM Pepstatin A; and IncA_87–246(polyA)_-His_6x_ was resuspended in 8.45 mM Potassium phosphate mono-basic/40.5 mM Sodium phosphate di-basic pH 7.4, 75 mM NaCl, 10% (w/v) glycerol, 0.5 mM TCEP-HCl, 1 mM PMSF, 10 µM Leupeptin, and 1.5 µM Pepstatin A. Resuspended bacteria were lysed by passage through an EmulsiFlex C3 high pressure homogenizer (Avestin). Lysates were then clarified by centrifugation at 35,000 rpm for 30 min at 4 °C, before being incubated with NiNTA beads for 1 h at 4 °C. The NiNTA beads were then loaded on to gravity chromatography columns and washed with resuspension buffer containing increasing amounts of imidazole (50 mM, 75 mM, and 100 mM). Proteins were eluted with resuspension buffer containing 400 mM imidazole and concentrated in centrifugal concentrators (3000 Dalton cutoff). Purified proteins were resolved on a HiLoad 16/60 Superdex prep-grade 200 column (GE Healthcare) equilibrated with their indicated buffers without glycerol containing 0.5 mM TCEP. The fractions containing protein were collected and concentrated. The purity of each protein was assessed by sodium dodecyl sulfate polyacrylamide gel electrophoresis (SDS-PAGE) and Coomassie Blue staining. Protein concentrations were determined by measuring the absorbance at 280 nm.

### Complementation of IncA KO *C. trachomatis* with IncA mutants

IncA KO *C. trachomatis* L2 and the IncA KO complemented with IncA_WT_-FLAG and IncA_(1–237)_-FLAG were previously generated^11^. To complement IncA KO *Chlamydia* with IncA-FLAG mutants, 5 x 10^7^ IncA KO elementary bodies and 10 µg of unmethylated DNA were mixed with 100 µl of 2x transformation buffer (20 mM Tris, pH 7.5, 100 mM CaCl_2_) and brought up to 200 µl with sterile water. Transformations were incubated at room temperature for 35 min and then mixed with 13 ml of cell culture medium (Dulbecco's Modified Eagle Medium (DMEM) supplemented with 10% calf serum, 2 mM l-glutamine, non-essential amino acids, 1 mM sodium pyruvate). Two milliliters of transformation was added to each well of a 6-well plate containing confluent Vero cells. Antibiotic selection was initiated at 18 hpi with 100 ng ml^−1^ chloramphenicol. At 46 hpi, the cells were lysed in sterile water and the lysate was used to infect four T_175_ flasks containing confluent Vero cells. The chloramphenicol concentration was increased to 200 ng ml^−1^ and 1 µg ml^−1^ cycloheximide was added. At 46 hpi, the cells were lysed in sterile water and the lysate was used to infect two T_175_ flasks containing confluent Vero cells. The chloramphenicol concentration was increased to 400 ng ml^−1^, and cycloheximide was maintained at 1 µg ml^−1^. At 72 hpi, the cell lysate was used to infect one T_175_ flask containing confluent Vero cells. The transformations were subsequently harvested every 46 hpi and the lysate was used to infect one T_25_ flask containing confluent Vero cells until the flask was 30%–60% infected. The selection medium contained 400 ng ml^−1^ chloramphenicol and 1 µg ml^−1^ cycloheximide for all remaining passages. Individual clones were isolated by limited dilution^30^ on Vero monolayers for 7–10 days in cell culture medium supplemented with 400 ng ml^−1^ chloramphenicol and 1 µg ml^−1^ cycloheximide. To confirm IncA-FLAG expression, cells were infected with clonal isolates for 24 h at 37^ o^C, fixed, labeled with anti-FLAG antibody and analyzed by immunofluorescence microscopy. IncA-FLAG expression was induced with 10 ng ml^−1^ anhydrotetracycline at 6 hpi. Selected clones were then purified by density gradient centrifugation^31^. Briefly, infected cells were harvested in K36 buffer (50 mM K_2_HPO_4_, 48.9 mM KH_2_PO_4_, 100 mM KCl, 14.9 mM NaCl, pH 7.0) and lysed by sonication. Lysates were clarified by centrifugation at 500 x *g* for 15 min at 4 °C and the supernatants were layered on top of a 30% renografin (Mallinckrodt) cushion, before being centrifuged at 40,000 x *g* for 30 min at 4 °C. Each pellet was then resuspended in K36 buffer and layered on top of a discontinuous renografin gradient consisting of 54% (bottom layer), 44% (middle layer), and 40% (top layer) renografin solutions. All renografin solutions were prepared in K36 buffer. The gradients were centrifuged at 40,000 x *g* for 1 h at 4 °C. The band at the 54%/44% interface was harvested, diluted in SPG (17.4 mM Na_2_HPO_4_, 2.6 mM NaH_2_PO_4_, 4.9 mM l-glutamic acid, pH 7.4) and pelleted by centrifugation at 40,000 x *g* for 30 min at 4 °C. The pellet was then resuspended in SPG and stored at −80 °C. A summary of all the *Chlamydia* strains used in this study is presented in Supplementary Table 3.

### Western blot

Infected cells were lysed 24 hpi by scraping into NuPage LDS sample buffer (Invitrogen) containing 2 µM pepstatin A, 10 µM leupeptin, 1 mM PMSF, 10 mM sodium fluoride, and 5.4 mM sodium orthovanadate. After boiling for 5 min at 95 ^o^C, samples were separated by SDS-PAGE and transferred to Polyvinylidene difluoride (PVDF) membrane at 100 V for 1 h. Membranes were incubated in blocking buffer [TBS-T (25 mM Tris, pH 7.5, 150 mM NaCl, 0.1% Tween-20) containing 3% bovine serum albumin] for 1 h at room temperature. Blots were then incubated with mouse anti-FLAG (Sigma, F1804) or goat anti-MOMP (Virostat, 1621) antibodies diluted in blocking buffer overnight at 4 °C. Following several washes with TBS-T, the blots were incubated with anti-mouse and anti-goat HRP-conjugated secondary antibodies (Pierce) diluted in TBS-T containing 0.5% low-fat milk powder for 1 h at room temperature. Blots were then washed several times with TBS-T and revealed using SuperSignal West Dura substrate (Pierce).

### Homotypic fusion assay

In all experiments, 6 x 10^4^ HeLa cells (ATCC) were seeded on glass coverslips 24 h prior to being infected with wild-type *C. trachomatis* or various *C. trachomatis* mutants at a MOI of 5 in DMEM containing 10% calf serum, 2 mM l-glutamine, 1 mM sodium pyruvate, non-essential amino acids, and 10 µg ml^−1^ gentamicin. The infection was synchronized by centrifugation for 1 h at 1000 x *g* (20 **°**C). The infected cells were then transitioned to 37 °C and 5% CO_2_ for the duration of the experiment. IncA-FLAG expression was induced at 6 h post infection (hpi) with 10 ng ml^−1^ (IncA_WT_-FLAG, IncA_1–237_-FLAG, IncA_1–243_-FLAG, IncA_1–246_-FLAG) or 20 ng ml^−1^ anhydrotetracycline (IncA_1–246(G144A)_-FLAG and IncA_1–246(polyA_-FLAG). At 24 hpi, the cells were fixed with ice-cold 100% methanol for 10 min and blocked in 25 mM Hepes pH 7.4, 150 mM NaCl, 10% horse serum, 0.9 mM calcium chloride, 0.5 mM magnesium chloride, 0.1% Triton X-100. Next, coverslips were stained with mouse anti-FLAG (Sigma, F180) and goat anti-MOMP (ViroStat, 1621) primary antibodies for 1 h at room temperature (RT). After several washes, the coverslips were incubated with secondary antibodies donkey anti-mouse Alexa Fluor 555 (Invitrogen) and donkey anti-goat Alexa Fluor 488 (Invitrogen) and 5 µg ml^−1^ Hoechst (Invitrogen) for 1 h at room temperature (RT). The coverslips were washed and mounted using ProLong Diamond AntiFade Mountant (Invitrogen). Single and multiple inclusions were quantified in a total of 250 infected cells for each *Chlamydia* strain. Images were acquired with a Nikon TiE inverted epi-fluorescence microscope equipped with a 60x oil-immersion objective and Nikon Elements AR software. The percentage of cells with single inclusions was calculated and normalized to either the IncA KO complemented with IncA_WT_-FLAG or IncA_1–246_-FLAG as described in the figure legend. Statistical significance was measured using a two-tailed student *t*-test.

### Heterotypic fusion (co-infection) assay

HeLa cells were co-infected with mCherry-expressing *Chlamydia* (L2_mCherry_, MOI 2) and IncA_mutant_-FLAG complemented-IncA KO *Chlamydia* (MOI 5), which constitutively expresses GFP. The infection was synchronized by centrifugation for 1 h at 1000 x *g* (20 **°**C). The infected cells were then transitioned to 37 °C and 5% CO_2_ for the duration of the experiment. IncA_mutant_-FLAG expression was induced at 5 hpi as described above. DMSO (non-induced), which is effectively the IncA KO, served as negative control. The cells were fixed 24 hpi with 4% paraformaldehyde for 20 min. DNA was labeled with 5 µg ml^−1^ Hoechst (Invitrogen) for 15 min at RT. Following several washes, the coverslips were mounted with ProLong Diamond AntiFade Mountant (Invitrogen). The percentage of WT inclusions (red) that fused with IncA_mutant_-FLAG inclusions (green) was enumerated. A total of 150 red WT inclusions for each co-infection condition were counted. Images were acquired with a Nikon TiE inverted epi-fluorescence microscope equipped with a 60x oil-immersion objective and Nikon Elements AR software. Data were normalized to IncA_WT_-FLAG complemented IncA KO *Chlamydia*. Statistical significance was measured using a two-tailed student *t*-test.

### Crystallographic methods

The proteolysis-resistant fragment IncA_87–246_ and the mutant protein IncA_87–246(G144A)_ were crystallized using the vapor diffusion hanging drop method by mixing 2 µl of purified protein (typically concentrated at 7.5 mg ml^−1^) with an equal volume of crystallization solution containing 0.2 M Sodium acetate trihydrate pH 8.0 and 20% (w/v) Polyethylene Glycol 3350. IncA_87–246_ crystals were harvested in nylon cryo-loops, cryo-protected with 27% ethylene glycol and flash-frozen in liquid nitrogen. For single-wavelength anomalous diffraction (SAD) phasing, IncA_87–246_ crystals were soaked for one minute in 5 µl of cryo-protectant solution supplemented with 0.5 M sodium iodide (NaI), and back-washed to remove the excess of NaI prior to plunging in liquid nitrogen. High-resolution diffraction data for native IncA_87–246_ were collected at beamline 9–2, at Stanford Synchrotron Radiation Lightsource (SSRL), on a Dectris Pilatus 6M detector. Crystals of IncA_87–246(G144A)_ and IncA_87–246_ soaked in NaI were diffracted on a Rigaku MicroMax-007 HF diffractometer equipped with a Pilatus3 R 200K direct detector. All steps of data indexing, integration, and reduction were carried out using HKL2000^32^, HKL3000^33^, and *CCP4* programs^34^. The structure was solved by SAD using an ultra-redundant dataset (i.e., overall redundancy >16) collected in-house. Sixteen iodine sites were located by *phenix.autosol*^35^ and used to calculate an initial set of SAD phases with Figure of Merit (FOM) equal to 0.37 between 15–1.8 Å resolution. An initial electron density map calculated with SAD phases was improved by solvent flattening and histogram matching, auto-built alternating cycles of automated model building using *phenix.autobuild*^36^ and manual rebuilding using COOT^37^. The completed model was then subjected to positional and anisotropic B-factor refinement to the highest resolution available (~ 1.12 Å) using *phenix.refine*^38^ and subjected to final re-refinement using PDB-redo^39^, which yielded the best *R*_work_/*R*_free_ and stereochemistry. The triclinic structure of IncA_1–246(G144A)_ was solved by molecular replacement using the wild-type IncA_1–246_ structure as the search model, as implemented in PHASER^40^. The best solution was subjected to positional and B-factor refinement in *phenix.refine*^38^ using all reflections between 15–1.95 Å resolution. The final models were refined to a *R*_work_/*R*_free_ of 14.1/16.8% (IncA_87–246_) and 16.9/21.2% (IncA_87–246(G144A)_) using all diffraction data between 15–1.12 Å and 15–1.95 Å, respectively. Final model validation was done using MolProbity^41,42^. Crystallographic data collection and refinement statistics are shown in Table 1. Ribbon diagrams and surface representations were prepared using the program PyMOL (The PyMOL Molecular Graphics System, Version 1.2r3pre, Schrödinger, LLC.). Intramolecular contacts were measured using the Pisa server^43^ and PDBsum^44^, and secondary structure superimpositions were carried out in Coot^37^.

### Analytical ultracentrifugation sedimentation velocity assay

AUC-SV analysis was carried out in a Beckman XL-A Analytical Ultracentrifuge. IncA samples were dissolved at 75 μM and 150 μM (corresponding to 1.5 and 3 mg ml^−1^) in 20 mM HEPES pH 7.5, 150 mM NaCl, 1 mM DTT and were spun at 45,000 rpm at 4 °C. Absorbance values between 285 nm were fit to a continuous sedimentation coefficient (c(s)) distribution model in SEDFIT^45^. Data were visualized and presented using GUSSI (University of Texas Southwestern Medical Center).

### Molecular dynamics simulations

Simulations of IncA_87–246_, IncA_87–237_, and IncA_87–246(G144A)_ were performed using NAMD2.12^46^ and the CHARMM36m protein forcefield^47^. To prepare these structures into models for equilibrium sampling, they were solvated with the TIP3P water model using the Solvate plugin in VMD^48^ and each system was charge-neutralized and ionized with NaCl to a concentration of 150 mM using VMD’s AutoIonize plugin. Each model was then subjected to 10,000 steps of gradient minimization. After minimization, harmonic restraints were applied to the protein backbone with a force constant of 5 kcal mol^−1^ during gradual thermalization of the system: the initial temperature was set to 50 K and then incrementally increased by 5 K every 10 ps. Once the systems reached 310 K, the last phase of model preparation was a gradual release of the restrained protein backbone atoms. Ten iterations were employed, removing 0.1 kcal mol^−1^ of the harmonic restraint’s force constant after intervals of 50 ps. Equilibrium simulations of the prepared models were run on the NCSA Blue Waters supercomputer and TACC Stampede2. Full electrostatics were calculated at every other timestep, while non-bonded interactions were calculated at every timestep. The Particle Mesh Ewald (PME) method was used to calculate long-range electrostatic interactions. Short-range non-bonded interactions were calculated within a cut-off of 12 Å. Bonds to hydrogen were constrained using the SHAKE and SETTLE algorithms for protein and solvent, respectively. Temperature regulation was accomplished using the Langevin thermostat method, with a damping coefficient of 1.0 ps^−1^. System pressure was maintained at 1.0 bar using the Nose-Hoover Langevin piston barostat. Each equilibrium simulation was run for 4 µs with a timestep of 2 fs. Trajectory analysis was conducted with VMD. Before processing, the three trajectories were aligned by minimizing the RMSD between backbone atoms of the initial equilibrated structure and all subsequent frames.

### Markov state model analysis

Markov State Model (MSM) construction and analysis were conducted using the MSMBuilder^49^ application. To prepare the IncA_87–246_ and IncA_87–237_ trajectories, each frame was aligned to the first in its series by RMSD between backbone atom coordinates. Water and ions were removed, and copies were saved every ten frames, with each frame in both of the over 40,000-frame datasets representing a 100 ps interval between samples. Prior to constructing MSMs, the trajectories were clustered by RMSD between atomic coordinates according to the *k*-medoids algorithm; loop regions were ignored. The initial choice of *k*-value was determined by a parameter sweep against the resultant within-cluster sum of squares, or inertia, as a metric of fit quality. The selection criterion for optimal *k* was based on the “Elbow method,” where returns on fit quality diminish steeply at *k* + 1. This pointed to an optimal value of *k* = 6. Owing to both the limitations of this approach and the sensitivity of the resulting MSMs to this parameter, the range of tested *k* values was *k* = {4,8}, followed by sparse sampling up to *k* = 4000 for both systems. After, MSMs were built from the clustered trajectories, testing differing model lag times using maximum likelihood estimation and (*N*_states_−1) dynamical timescales for different input *k*-clusters. The resulting models of IncA_87–246_ and IncA_87–237_ were scored based on their generalized matrix Rayleigh quotient^20^ (GMRQ) training and testing scores. The optimal lag time (*τ*) for both IncA_87–246_ and IncA_87–237_ models was determined to be *τ* = 10 ns and was used for all reported results. For the MSMs shown in Supplementary Fig. 9, panels (a) and (b): *k* = 6 clustered input trajectories for IncA_87–237_ and IncA_87–246_; panel c: *k* = 500 clustered input trajectory for IncA_87–246_ only. Free energies were calculated according to $\Delta {G} = - {k}_{\mathrm{b}}{T}\;{\mathrm{log}}\;{p}$, where *k*_b_ is the Boltzmann constant (kcal mol^−1^) and *p* is the estimated equilibrium population of a given Markov state.

### Thermal stability assay

The thermal stability of IncA_87–246_, IncA_87–246(G144A)_, and IncA_87–246(polyA)_ was measured by recording changes in the ellipticity intensity at 220 nm as a function of temperature. Circular dichroism (CD) spectra were recorded using a Jasco J-810 spectropolarimeter equipped with a Neslab RTE7 refrigerated recirculator available at the Sidney Kimmel Cancer Center X-ray Crystallography and Macromolecular Characterization Shared Resource. IncA samples were dissolved in 20 mm sodium phosphate (pH 7.4), 100 mm NaCl and 1 mM DTT at a final concentration of 1.0 μM in a 1 cm rectangular quartz cuvette (Starna Cells, Inc.). CD spectra were recorded between 195 nm and 260 nm. The variations in ellipticity at 220 nm as a function of temperature in 1 °C increments were measured over the range 20−75 °C. Slow cooling to 20 °C followed by a CD scan for secondary structure demonstrated that the unfolding of all IncA samples are largely irreversible. The apparent Melting Temperature (*app*Tm) values for IncA_87–246_, IncA_87–246(G144A)_, and IncA_87–246(polyA)_ are 38, 38.5, and 38.5 °C, respectively.

### Reporting summary

Further information on research design is available in the Nature Research Reporting Summary linked to this article.

## Supplementary information

Supplementary information

Description of Additional Supplementary Files

Supplementary Movie 1

Supplementary Movie 2

Supplementary Movie 3

Supplementary Movie 4

Reporting Summary

Source data

## Data Availability

The atomic coordinates and structure factors for wild-type IncA_87–246_ and IncA_87–246(G144A)_ have been deposited in the Protein Data Bank with accession codes 6E7E and 6E6A, respectively. The authors declare that all other relevant data supporting the findings of this study are included in this published article and its Supplementary Information files, or from the corresponding authors upon request.
