# Supplementary material for: Structural basis for the homotypic fusion of chlamydial inclusions by the SNARE-like protein IncA
Source: Nat Commun. 2019 Jun 21;10:2747. doi: 10.1038/s41467-019-10806-9 (PMC6588587; doi:10.1038/s41467-019-10806-9)
Supplement: Supplementary file 1 — Supplementary information [file 41467_2019_10806_MOESM1_ESM.pdf]

SUPPLEMENTARY INFORMATION

Structural basis for the homotypic fusion of chlamydial inclusions by the SNARE-like protein IncA

Cingolani, G. et al.

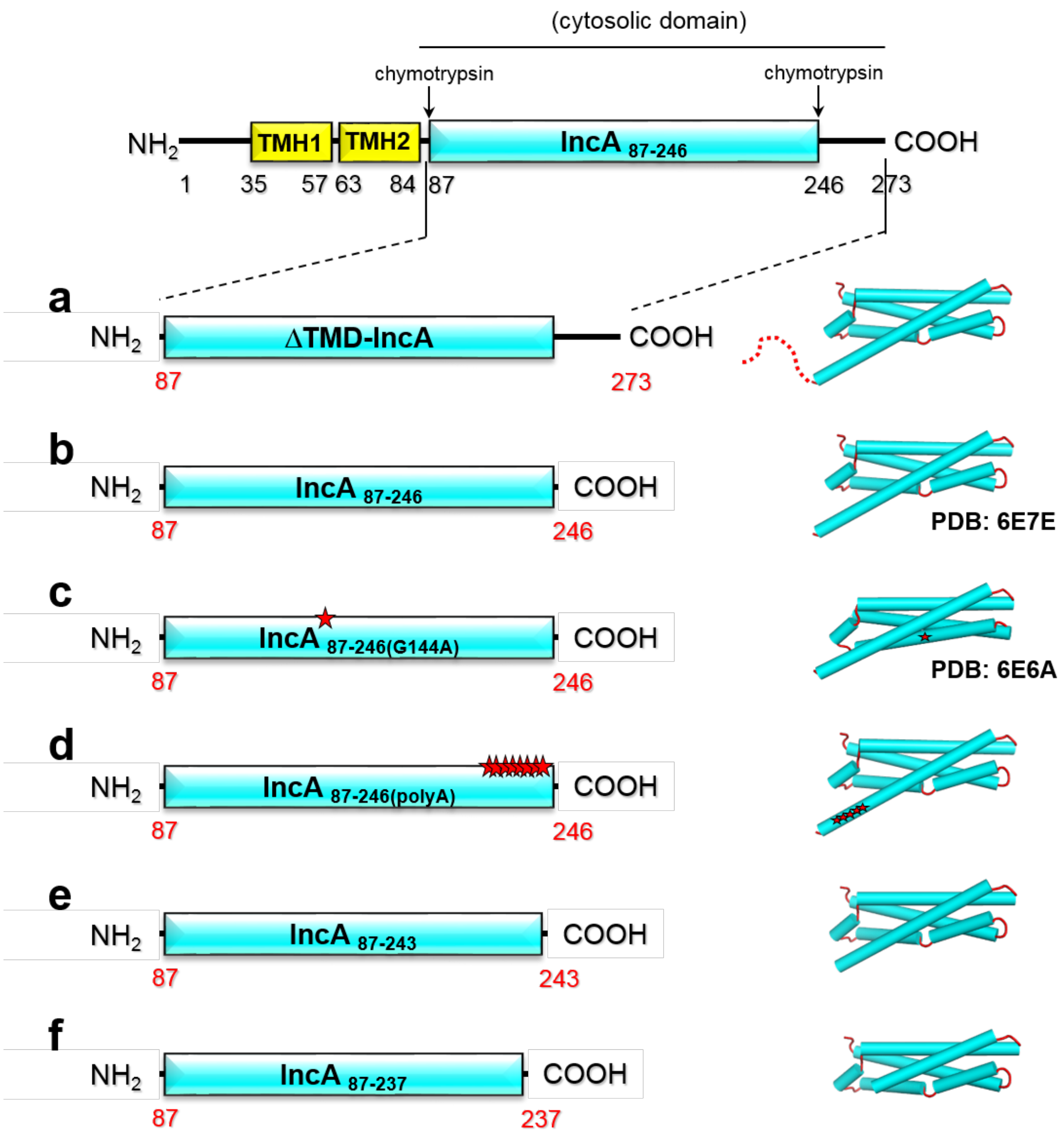

Supplementary Figure 1. Schematic diagram of $IncA_{WT}$ and soluble $IncA_{mutant}$ constructs used in this study. The crystal structures of IncA soluble fragments determined experimentally are denoted by a PDB ID. (**a**) depicts ΔTMD-IncA in which the transmembrane domain was removed, thus creating soluble IncA; (**b**) depicts $IncA_{87\text{-}246}$, which was crystallized (PDB: 6E7E); (**c**) depicts $IncA_{87\text{-}246(G144A)}$, which was crystallized (PDB: 6E6A); (**d**) depicts $IncA_{87\text{-}246(polyA)}$ in which 8 residues were mutated to alanines; (**e**) depicts $IncA_{87\text{-}243}$, and (**f**) depicts $IncA_{87\text{-}237}$.

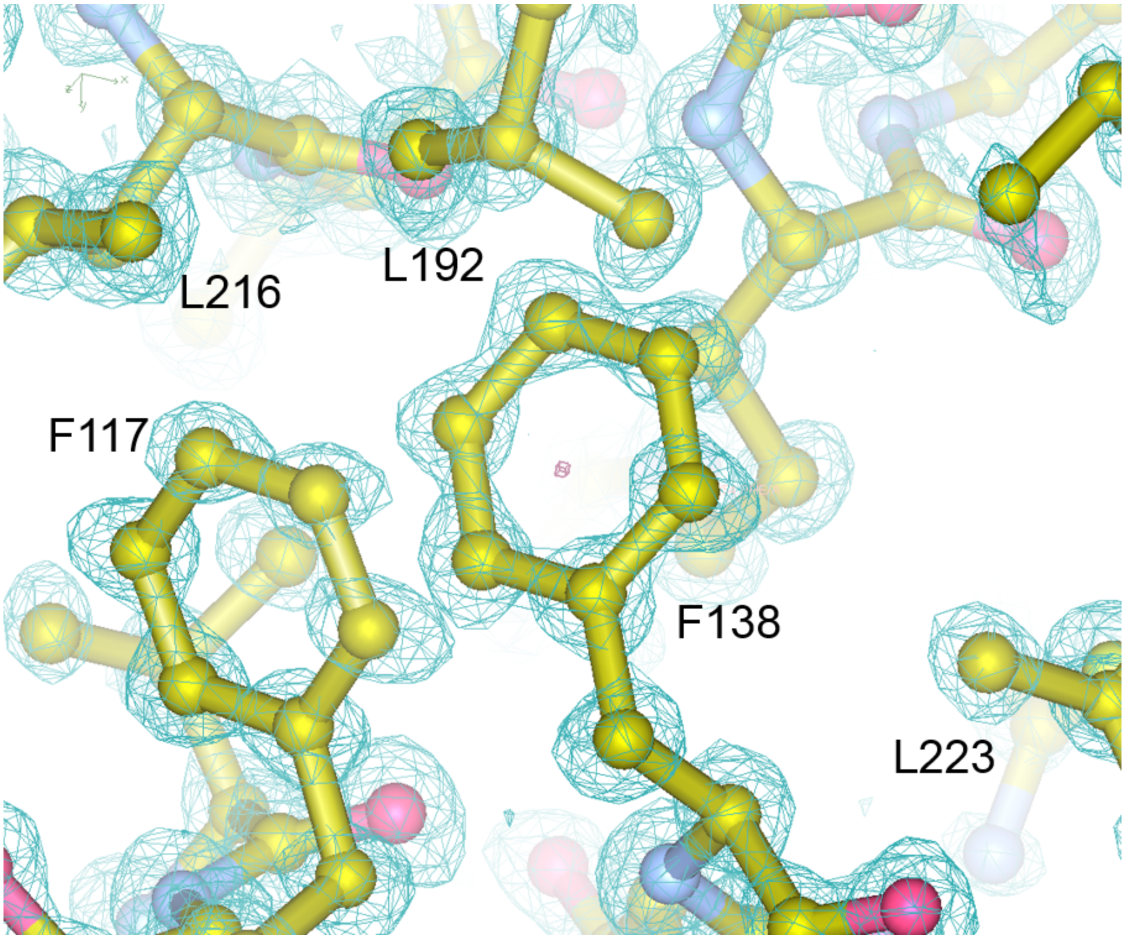

Supplementary Figure 2. Magnified view of IncA$_{87\text{-}246}$ final 2Fo-Fc electron density map calculated at 1.12 Å resolution and overlaid to the final atomic model (shown as sticks). The electron density is colored in cyan and contoured at 1.9 σ above background. The figure was generated using Coot[1].

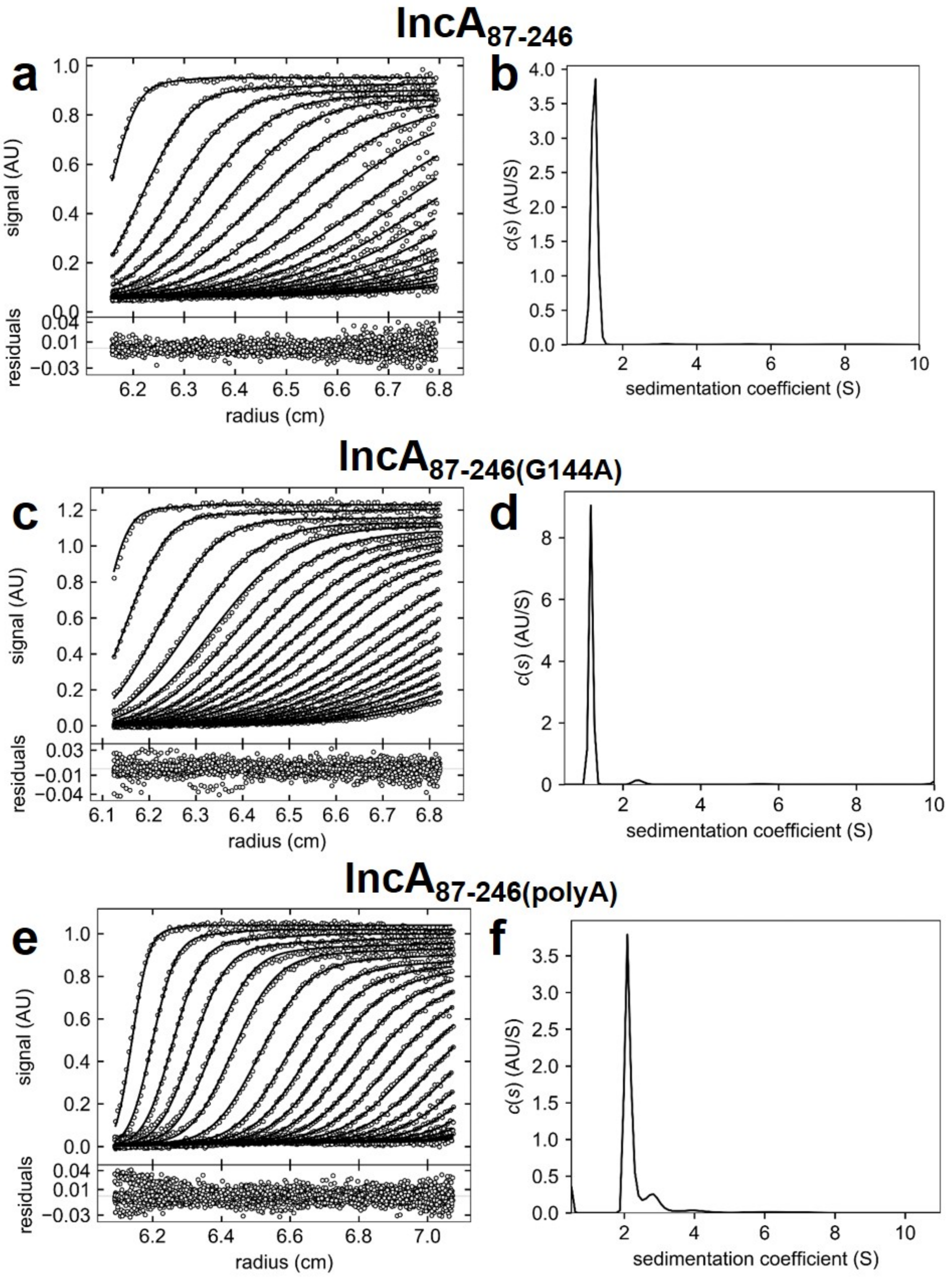

Supplementary Figure 3: Analytical Ultracentrifugation Sedimentation Velocity (AUC-SV). Panels (**a**), (**c**) and (**e**) show the position of the sedimentation boundary during AUC-SV analysis for IncA$_{87\text{-}246}$, IncA$_{87\text{-}246(G144A)}$ and IncA$_{87\text{-}246(polyA)}$, respectively. IncA$_{87\text{-}246}$ and IncA$_{87\text{-}246(polyA)}$ were analyzed at ~3.0 mg ml^{-1} and IncA$_{87\text{-}246(G144A)}$ was at ~1.5 mg ml^{-1}. In all three cases, the sedimentation boundary exhibits monophasic behavior, which is indicative of a single major component in solution, migrating with apparent sedimentation coefficients of 1.240S (**b**), 1.178S (**e**) and 2.131S (**f**). Conversion of the distribution of the apparent sedimentation coefficient to molecular mass revealed a M.W. of ~19.3 kDa (IncA$_{87\text{-}246}$), ~19.3 kDa (IncA$_{87\text{-}246(G144A)}$) and ~58.4 kDa (IncA$_{87\text{-}246(polyA)}$) (**Supplementary Table 1**).

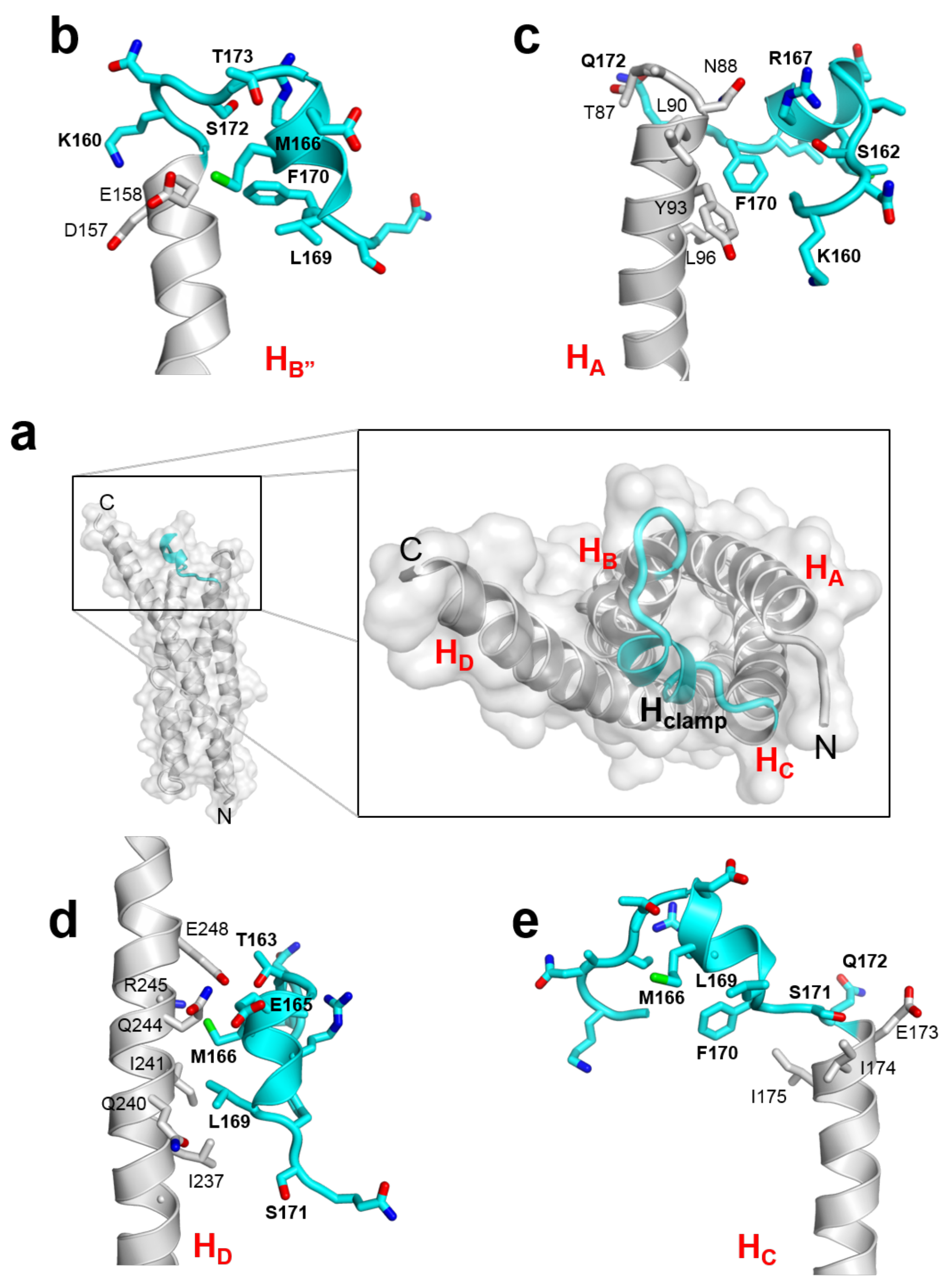

Supplementary Figure 4. Identification of the contacts made by H_{clamp} and the α-helices $H_{B"}$, H_A, H_D, and H_C. Central panel (**a**) shows side and top views of $IncA_{87-246}$ with H_{clamp} colored in cyan and the rest of IncA in light gray. The ribbon diagram is overlaid to a semitransparent solvent surface. Panels (**b**) through (**e**) show magnified views of the individual contacts that the clamp makes with helices $H_{B"}$ (**b**), H_A (**c**), H_D (**d**), and H_C (**e**). **Supplementary Table 2** lists the number of contacts and overall energetics of binding.

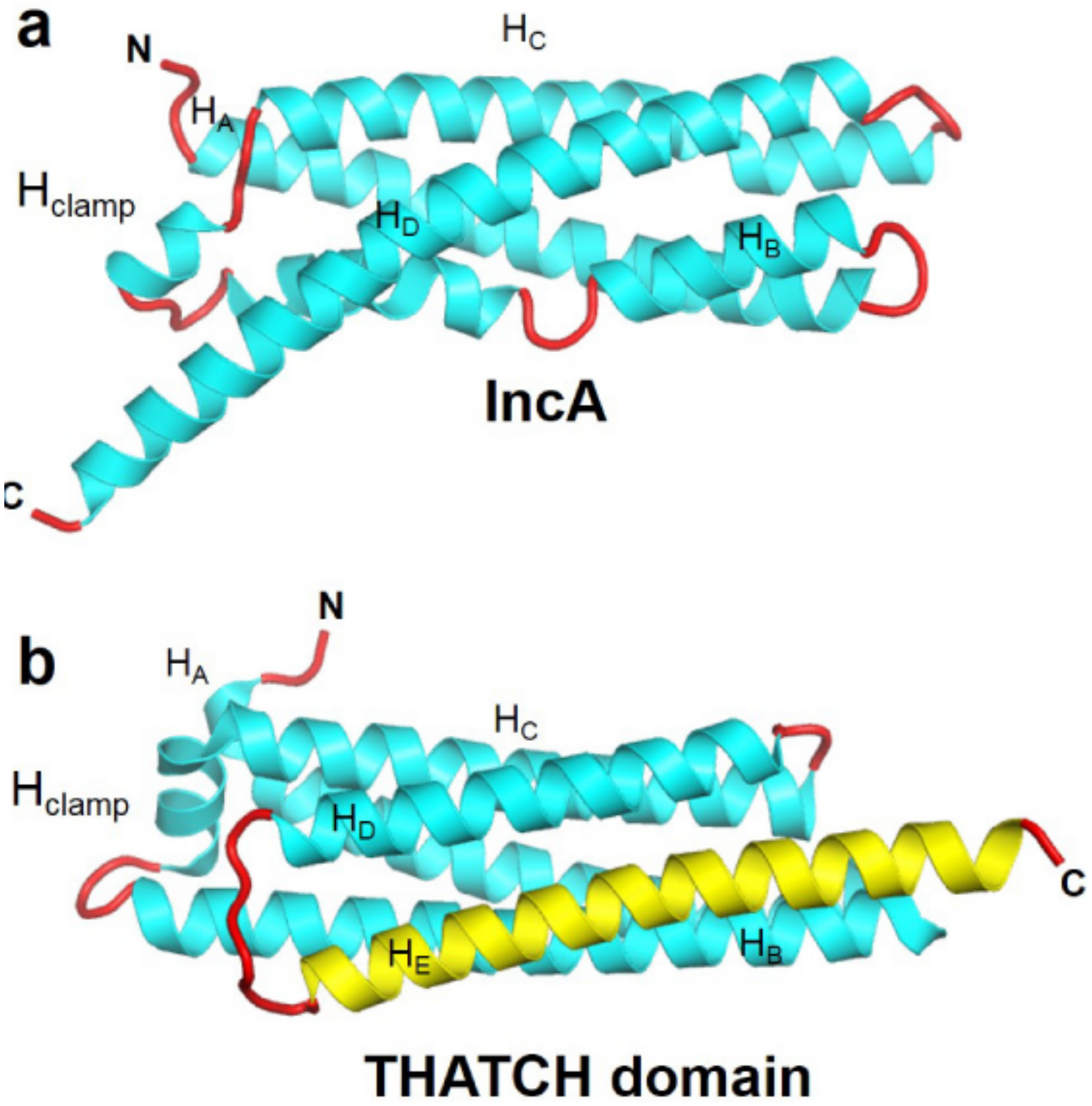

Supplementary Figure 5. Ribbon diagrams of (a) IncA$_{87\text{-}246}$ and (b) HIP1R thatch domain core (PDB id 1R0D). The two crystallographic models were refined to a resolution of 1.12 Å and 1.75 Å, respectively. In both panels, the non-canonical four-helix bundle formed by helices H_A-H_D and H_{clamp} is colored in cyan and red while HIP1R fifth helix, H_E, is in yellow (see panel **b**). The two four-helix bundles superimpose with an RMSD ~ 2.11 Å, while the overall Cα RMSD is ~3.60 Å.

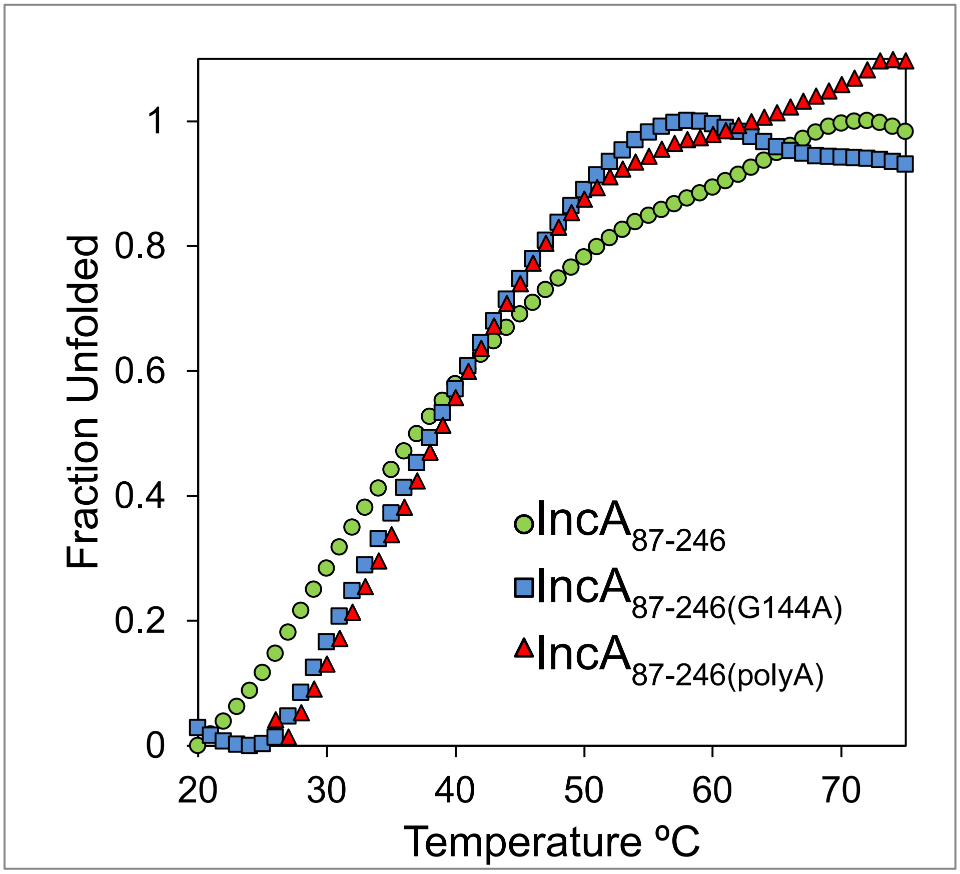

Supplementary Figure 6. IncA$_{87\text{-}246}$, IncA$_{87\text{-}246(G144A)}$, and IncA$_{87\text{-}246(polyA)}$ have comparable structural stability. For each construct, the fraction unfolded was estimated by measuring changes in the ellipticity intensity at 220 nm as a function of temperature. The apparent Melting Temperature (*app*Tm) values for IncA$_{87\text{-}246}$, IncA$_{87\text{-}246(G144A)}$, and IncA$_{87\text{-}246(polyA)}$ are 38, 38.5 and 38.5 °C, respectively.

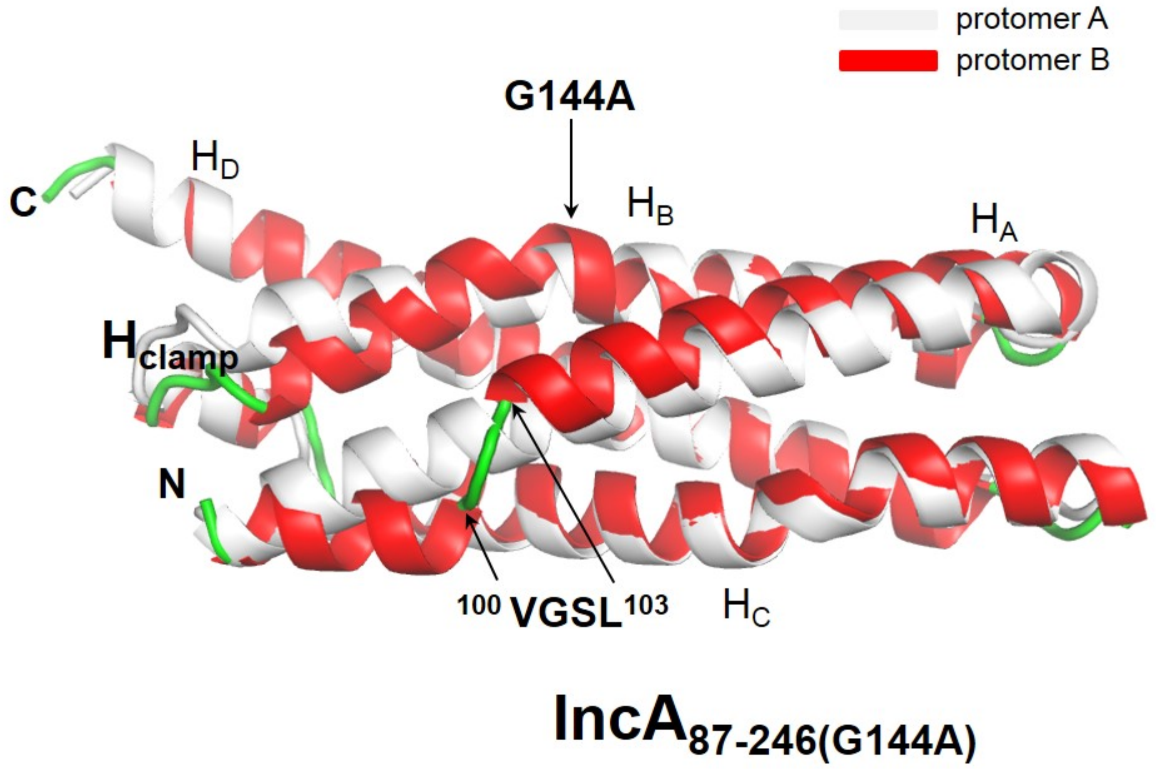

Supplementary Figure 7. Secondary structure superimposition of the two $IncA_{87\text{-}246(G144A)}$ protomers found in the triclinic unit cell. Protomer A is colored in gray, while protomer B is in red and green. The main structural differences are found in residues ^{100}VGSL^{103} of protomer B, at the base of helix A, as detailed in the main text.

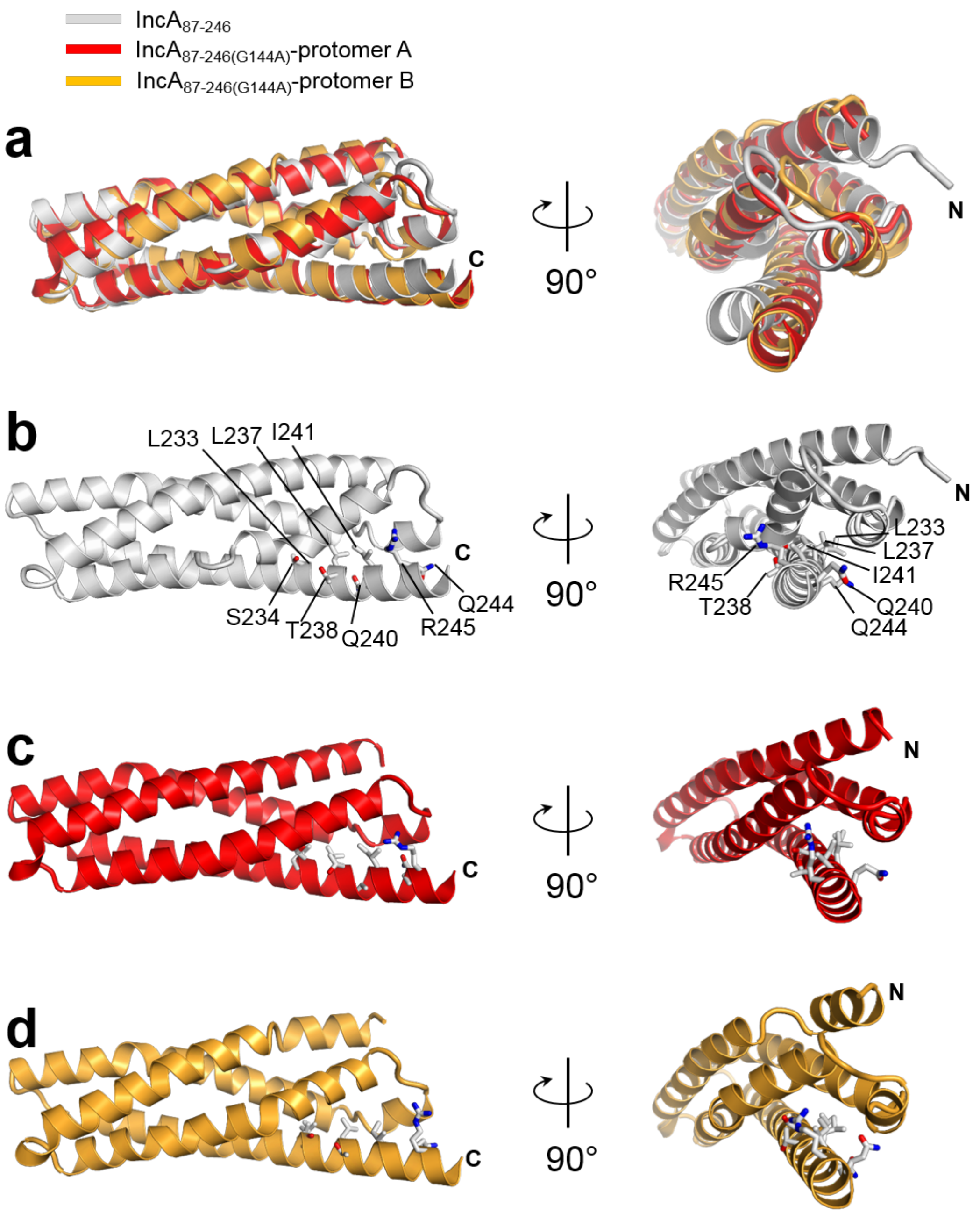

Supplementary Figure 8. Superimposition shows how the eight residues mutated in IncA$_{87\text{-}246(\text{polyA})}$ are oriented with respect to IncA$_{87\text{-}246(\text{G144A})}$ protomers, aligned against IncA$_{87\text{-}246}$. A structural alignment of IncA$_{87\text{-}246}$, IncA$_{87\text{-}246(\text{G144A})}$-protomer A, and IncA$_{87\text{-}246(\text{G144A})}$-protomer B is in panel (**a**). The three individual IncA constructs are shown in panels (**b**), (**c**), and (**d**), respectively. The eight residues mutated to alanine in IncA$_{87\text{-}246(\text{polyA})}$ are labeled in panel (**b**) and also shown as sticks in panels (**c**) and (**d**).

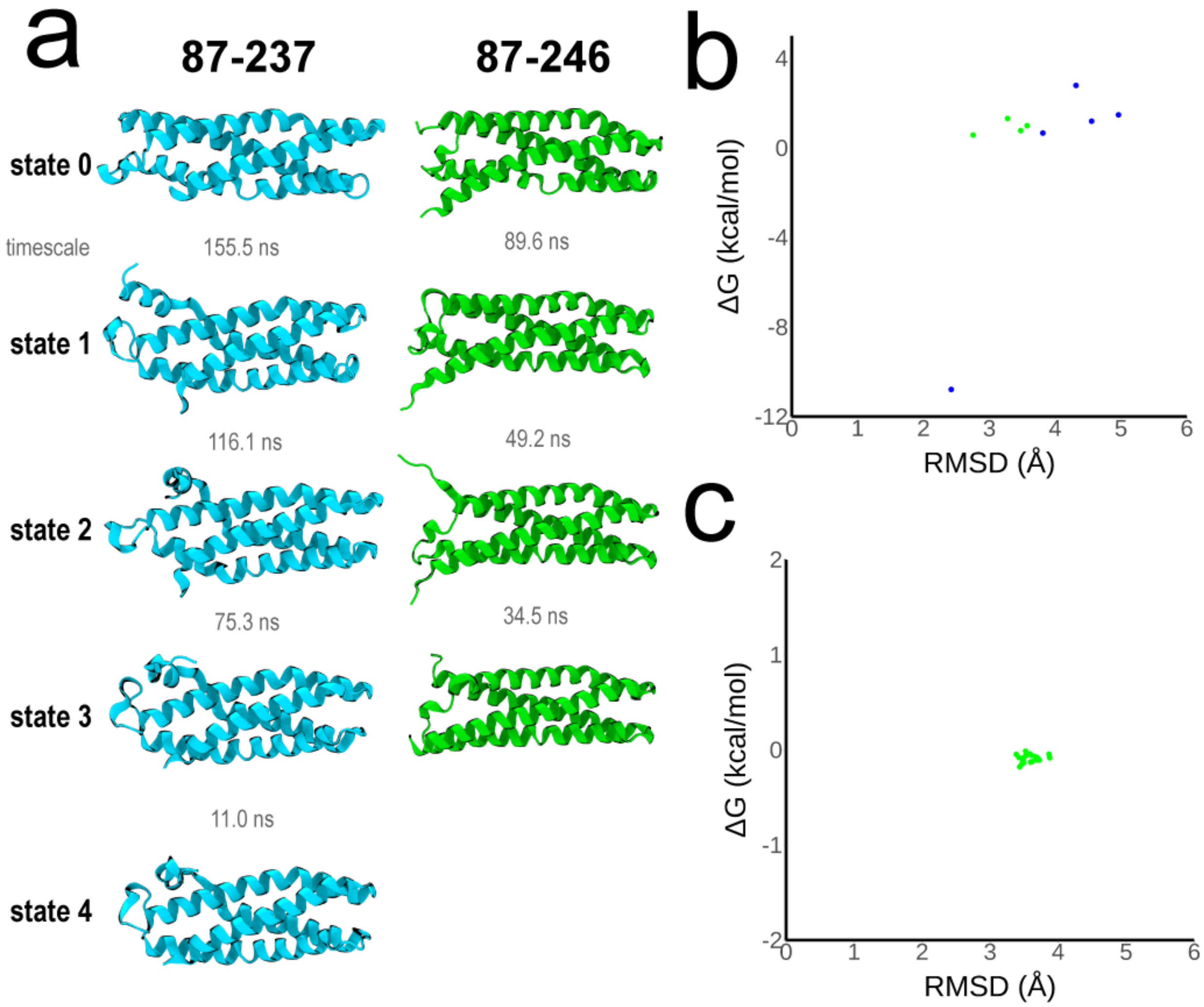

Supplementary Figure 9. Markov State Models (MSM) of IncA$_{87\text{-}246}$ and IncA$_{87\text{-}237}$ (**a**) Side-by-side comparison of the Markov states of IncA$_{87\text{-}246}$ (green) and IncA$_{87\text{-}237}$ (blue). These models were constructed from k=6 clustered input trajectories, loops were not considered for the MSM analysis. Pictures are in order the top scoring models of IncA$_{87\text{-}237}$. (**b**) Free energy landscape of the Markov states shown in panel a (colored accordingly). (**c**) Free energy landscape of the highest scoring IncA$_{87\text{-}246}$ MSM, constructed from a k=500 clustered input trajectory.

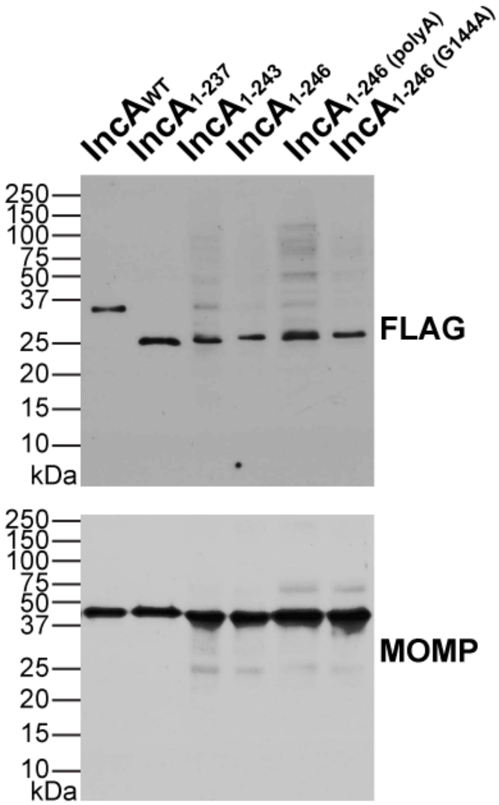

Supplementary Figure 10. Analysis of $IncA_{mutant}$ expression. Uncropped Western blots from Figure 4a. HeLa cells were infected with the indicated $IncA_{mutant}$-FLAG complemented IncA KO strains for 24 h and then lysed in sample buffer. Samples analyzed by Western blot using anti-FLAG and anti-*C. trachomatis* MOMP primary antibodies. Source data are provided as a Source Data file.

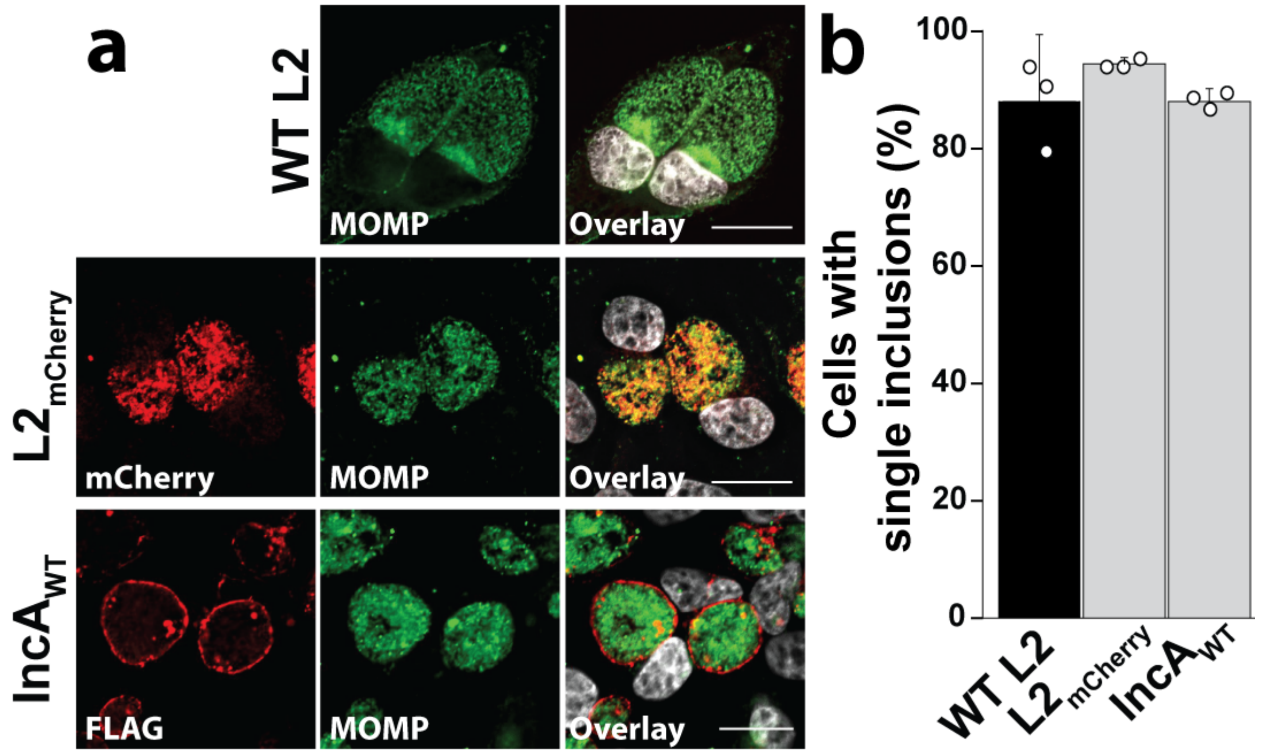

Supplementary Figure 11. WT *Chlamydia*, *Chlamydia* KO-complemented with IncA$_{WT-FLAG}$, and *Chlamydia* expressing mCherry (L2$_{mCherry}$) undergo comparable levels of homotypic fusion. **(a**) Immunofluorescence microscopy analysis of HeLa cells infected with the indicated wild type IncA strain (WT L2 and L2$_{mCherry}$) or IncA$_{WT}$-FLAG complemented IncA KO strain at 24hpi. Bacteria were labeled with anti-MOMP (green) antibody. DNA was stained with Hoechst (white). L2$_{mCherry}$ is indicated in red (middle panel). The expression of IncA$_{WT}$-FLAG on the inclusion was revealed with anti-FLAG antibody (red, lower panel). The ring-like FLAG staining shows that IncA$_{WT}$-FLAG is secreted on the inclusion membrane. Scale bar = 20µm. Images are representative of three independent experiments. **(b)** Quantification of homotypic fusion for wild type *Chlamydia* (WT L2), L2$_{mCherry}$, or IncA$_{WT}$-FLAG complemented IncA KO strain. The graph displays the means of three independent experiments ± the standard deviation. There is no statistical significance between strains (two-tailed student t-test). Source data are provided as a Source Data file.

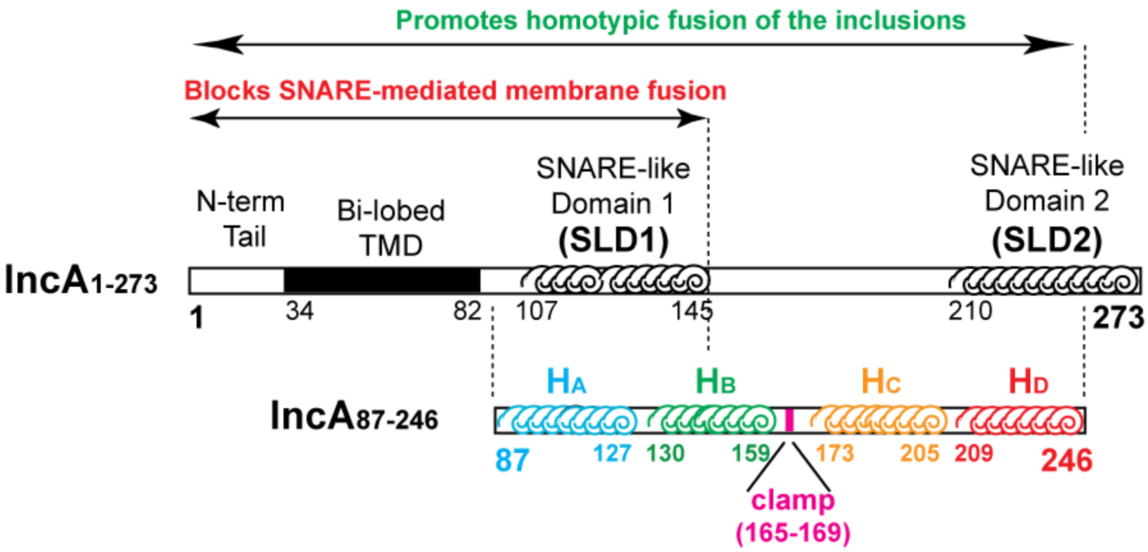

Supplementary Figure 12. Schematic diagram showing how the α-helices observed in the crystal structure of IncA correspond to the SNARE-like Domains 1 and 2 (SLD1 and SLD2) previously described for IncA[2, 3].

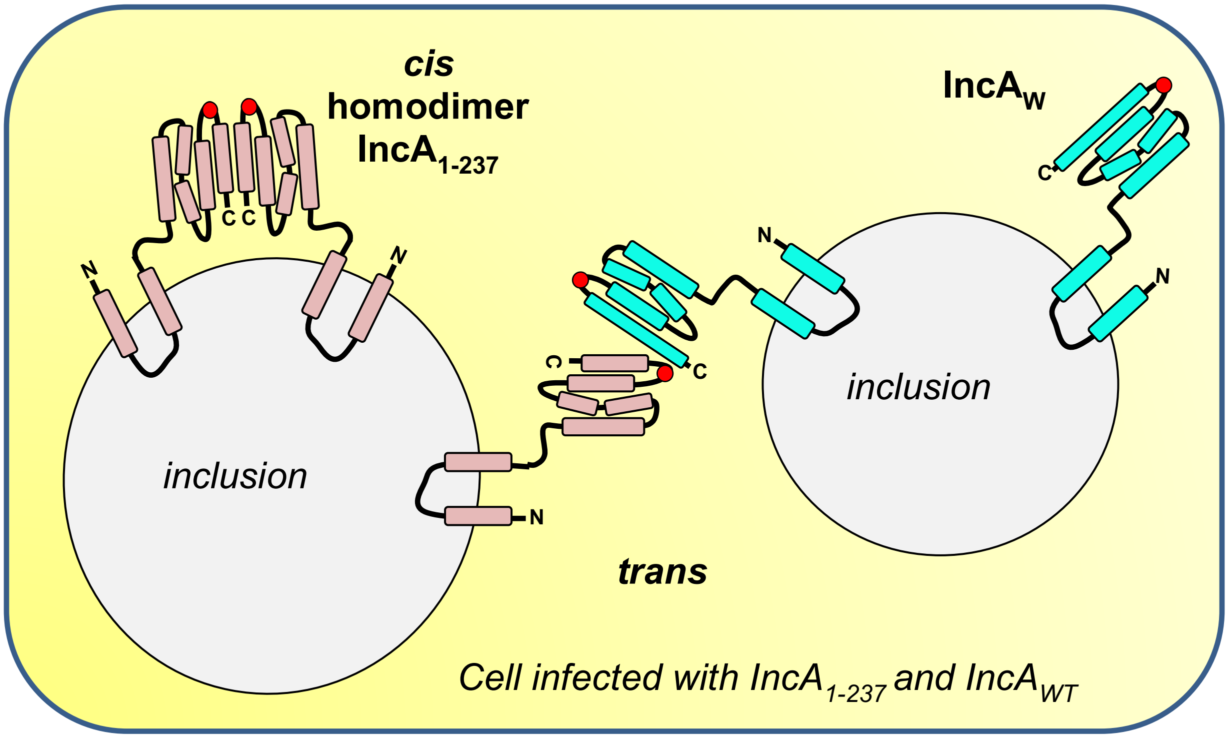

Supplementary Figure 13. Hypothetical mechanism of fusion for cells infected by *Chlamydia* that expresses $IncA_{1\text{-}237}$ (inclusion #1) and $IncA_{WT}$ (inclusion #2). The shorter $IncA_{1\text{-}237}$ is trapped in a high order oligomeric conformation (cis-homodimer in the illustration), which is incompetent for membrane fusion, whereas $IncA_{WT}$ is monomeric and competent for fusion. Complete rescue of $IncA_{1\text{-}237}$ loss of function is achieved by the formation of trans-heterodimers with $IncA_{WT}$.

Supplementary Table 1. Biophysical parameters measured using AUC-SV

	**IncA$_{87\text{-}246}$**	**IncA$_{87\text{-}246(G144A)}$**	**IncA$_{87\text{-}246(polyA)}$**
Protein Concentration (mg ml^{-1})	3.0	1.5	3.0
Apparent Sedimentation Coef., s (S)	1.240	1.178	2.131
Absolute Sedimentation Coef., $s_{20,w}$ (S)	1.289	1.225	2.257
Estimated / Theoretical M.W. (kDa)	19.3 / 19.7	19.3 / 19.7	58.4 / 19.3
Frictional Ratio, f/f_0	1.971	2.076	2.367

Supplementary Table 2. Interfaces and contacts made by IncA H_{clamp} residues with α-helices H_A, $H_{B''}$, H_C, and H_D

	H_{clamp}:H_A	H_{clamp}:$H_{B''}$	H_{clamp}:H_C	H_{clamp}:H_D
Interface area in (Å^2) *	236.3	295.3	112.5	218.8
Solvation Free Energy (ΔiG) (kcal/M) **	-3.3	-4.3	-1.2	-4.8
No. of hydrogen bonds ***	1	2	4	1
No. of non-bonded contacts ***	18	40	16	20

* Interface area (Å^2) calculated by Pisa[4] as the difference in total accessible surface areas of isolated and interfacing structures divided by two.
** Solvation Free Energy (ΔiG) gain upon formation of the interface (in kcal M^{-1}). Negative ΔiG in Pisa[4] corresponds to hydrophobic interfaces, or positive protein affinity.
*** Number of hydrogen bonds, nonbonded contacts and salt bridges calculated by PDBSum[5].

Supplementary Table 3. Recombinant *Chlamydia* strains used in this study

Name	Genetic background	Plasmid	Antibiotic resistance	Ref
$L2_{mCherry}$	L2 434/Bu	pBomb4-MCI	penicillin	[6]
IncA KO (CT119::bla)	L2 434/Bu	pACT-IncA	penicillin	[7]
$IncA_{WT}$-FLAG	CT119::bla	pBomb3-Tet-$IncA_{1-273}$-FLAG	penicillin and chloramphenicol	[7]
$IncA_{1-237}$-FLAG	CT119::bla	pBomb3-Tet-$IncA_{1-237}$-FLAG	penicillin and chloramphenicol	[7]
$IncA_{1-243}$-FLAG	CT119::bla	pBomb3-Tet-$IncA_{1-243}$-FLAG	penicillin and chloramphenicol	here
$IncA_{1-246}$-FLAG	CT119::bla	pBomb3-Tet-$IncA_{1-246}$-FLAG	penicillin and chloramphenicol	here
$IncA_{1-246(polyA)}$-FLAG	CT119::bla	pBomb3-Tet-$IncA_{1-246\ (polyA)}$-FLAG	penicillin and chloramphenicol	here
$IncA_{1-246(G144A)}$-FLAG	CT119::bla	pBomb3-Tet-$IncA_{1-246\ (G144A)}$-FLAG	penicillin and chloramphenicol	here

Supplementary Table 4. Primers used in this study

Primer	Sequence
FO515	CATGCCATGGCCACCGCTAATCTACATCTATAC
FO516	CCGCTCGAGAAGAGTTTTAGAAAGTTG
FO818	GAATTCGCGGCCGCATGGCAACGCCTACTCTAATCG
FO1023	CAATTCGCGGCCGCATGGCAACGCCTACTCTAATCG
FO1024	GAATTCGTCGACCTACTTGTCGTCATCGTCTTTGTAGTCCTCTTTTCG TTGTAATGCAATTTG
FO1067	GATTTTTATTCTTGTTTGCAAGCATTTAGAGATAACTATAAAGGTTTTG
FO1068	CCTTTATAGTTATCTCTAAATGCTTGCAAACAAGAATAAAAATCTTGA
FO1116	GATTCCCATGGCCACCGCTAATCTACATCTATACCAGG
FO1143	GAATTCGTCGACCTACTTGTCGTCATCGTCTTTGTAGTCTAATGCAAT TTGACTGG
FO1184	GAATTCGTCGACCTACTTGTCGTCATCGTCTTTGTAGTCTTTGGCCG CTAATGCCGC
FO1185	GAATTCCTCGAGTTTGGCCGCTAATGC

Supplementary Table 5. Plasmids used in this study

Plasmid	Plasmid Name
FD578	pET28a IncA$_{87\text{-}246}$-His$_{6x}$
FD915	pET28a IncA$_{87\text{-}246(G144A)}$-His$_{6x}$
FD923	pBOMB3
FD929	pBOMB3-Tet
FD930	pBOMB3-Tet IncA$_{1\text{-}246}$-FLAG
FD944	pBOMB3-Tet IncA$_{1\text{-}243}$-FLAG
FD945	pBOMB3-Tet IncA$_{1\text{-}246(G144A)}$-FLAG
FD948	pBOMB3-Tet IncA$_{1\text{-}246(polyA)}$-FLAG
FD985	pET28a IncA$_{87\text{-}246(polyA)}$-His$_{6x}$

SUPPLEMENTARY REFERENCES

1. Emsley P, Cowtan K. Coot: model-building tools for molecular graphics. *Acta Crystallogr D Biol Crystallogr* **60**, 2126-2132 (2004).

2. Paumet F*, et al.* Intracellular bacteria encode inhibitory SNARE-like proteins. *PloS One* **4**, e7375 (2009).

3. Ronzone E, Paumet F. Two coiled coil domains of Chlamydia trachomatis IncA affect membrane fusion events during infection. *PloS One* **8**, e69769 (2013).

4. Krissinel E, Henrick K. Inference of macromolecular assemblies from crystalline state. *J Mol Biol* **372**, 774-797 (2018).

5. Laskowski R, Jabłońska J, Pravda L, Vařeková R, Thornton J. PDBsum: Structural summaries of PDB entries. *Protein Sci* **27**, 129-134 (2018).

6. Bauler L, Hackstadt T. Expression and targeting of secreted proteins from Chlamydia trachomatis. *J Bacteriol* **196**, 1325-1334 (2014).

7. Weber M*, et al.* A Functional Core of IncA Is Required for Chlamydia trachomatis Inclusion Fusion. *J Bacteriol* **198**, 1347-1355 (2016).
