# Supplementary material for: Structural basis for the homotypic fusion of chlamydial inclusions by the SNARE-like protein IncA
Source: Nat Commun. 2019 Jun 21;10:2747. doi: 10.1038/s41467-019-10806-9 (PMC6588587; doi:10.1038/s41467-019-10806-9)
Supplement: Supplementary file 2 — Description of Additional Supplementary Files [file 41467_2019_10806_MOESM2_ESM.pdf]

Description of Additional Supplementary Files

File Name: Supplementary Movie 1
Description: (“lc_comp_final.mp4”, resolution: 2700x3000): Individual composition of the IncA87-246 trajectory, shown from same two angles as above.

File Name: Supplementary Movie 2
Description: (“sc_comp_final.mp4”, resolution: 2700x3000): individual composition of the IncA87-237 trajectory, shown from same two angles as above.

File Name: Supplementary Movie 3
Description: (“all3_comp_final.mp4”, resolution: 2650x882): Composition showing side-by-side comparison of all three IncA simulations performed: 87-246 (green), 87-237 (blue), G144A (orange). Each trajectory is represented via two viewing angles.

File Name: Supplementary Movie 4
Description: (“g1_comp_final.mp4”, resolution: 2700x3000): individual composition of the IncA87-246(G144A) trajectory, shown from same two angles as above.
