# Supplementary material for: Structural basis for the homotypic fusion of chlamydial inclusions by the SNARE-like protein IncA
Source: Nat Commun. 2019 Jun 21;10:2747. doi: 10.1038/s41467-019-10806-9 (PMC6588587; doi:10.1038/s41467-019-10806-9)
Supplement: Supplementary file 7 — Reporting Summary [file 41467_2019_10806_MOESM7_ESM.pdf]

nature research

Corresponding author(s): Fabienne Paumet, Gino Cingolani

Last updated by author(s): 2019/05/10

Reporting Summary

Nature Research wishes to improve the reproducibility of the work that we publish. This form provides structure for consistency and transparency in reporting. For further information on Nature Research policies, see Authors & Referees and the Editorial Policy Checklist.

Statistics

For all statistical analyses, confirm that the following items are present in the figure legend, table legend, main text, or Methods section.

n/a	Confirmed	
☐	☒	The exact sample size (*n*) for each experimental group/condition, given as a discrete number and unit of measurement
☐	☒	A statement on whether measurements were taken from distinct samples or whether the same sample was measured repeatedly
☐	☒	The statistical test(s) used AND whether they are one- or two-sided<br>*Only common tests should be described solely by name; describe more complex techniques in the Methods section.*
☒	☐	A description of all covariates tested
☒	☐	A description of any assumptions or corrections, such as tests of normality and adjustment for multiple comparisons
☐	☒	A full description of the statistical parameters including central tendency (e.g. means) or other basic estimates (e.g. regression coefficient) AND variation (e.g. standard deviation) or associated estimates of uncertainty (e.g. confidence intervals)
☒	☐	For null hypothesis testing, the test statistic (e.g. *F*, *t*, *r*) with confidence intervals, effect sizes, degrees of freedom and *P* value noted<br>*Give P values as exact values whenever suitable.*
☒	☐	For Bayesian analysis, information on the choice of priors and Markov chain Monte Carlo settings
☒	☐	For hierarchical and complex designs, identification of the appropriate level for tests and full reporting of outcomes
☒	☐	Estimates of effect sizes (e.g. Cohen's *d*, Pearson's *r*), indicating how they were calculated

Our web collection on statistics for biologists contains articles on many of the points above.

Software and code

Policy information about availability of computer code

Data collection

High-resolution diffraction data for native IncA87-246 were collected at beamline 9-2, at Stanford Synchrotron Radiation Lightsource (SSRL), on a Dectris Pilatus 6M detector.
Equilibrium simulations of the prepared models were run on the NCSA Blue Waters supercomputer and TACC Stampede2.
Nikon TiE inverted fluorescence microscope was used for image acquisition.

Data analysis

All steps of data indexing, integration, and reduction were carried out using HKL200033, HKL300034, and CCP4 programs. The structure was solved by single-wavelength anomalous diffraction (SAD) using an ultra-redundant dataset collected in-house. Iodine sites were located by phenix.autosol and used to calculate an initial set of SAD phases with Figure of Merit (FOM) equal to 0.37 between 15-1.8Å resolution. An initial electron density map calculated with SAD phases was improved by solvent flattening and histogram matching, auto-built alternating cycles of automated model building using phenix.autobuild and manual rebuilding using COOT. The completed model was subjected to positional and anisotropic B-factor refinement using phenix.refine and subjected to final re-refinement using PDB-redo. The triclinic structure of IncA1-246(G144A) was solved by molecular replacement using the wild-type IncA1-246 structure as the search model, as implemented in PHASER. Final model validation was done using MolProbity. Ribbon diagrams and surface representations were prepared using the program Pymol (The PyMOL Molecular Graphics System). Intramolecular contacts were measured using PDBsum, and secondary structure superimpositions were carried out in Coot.

Simulations of IncA87-246, IncA87-237, and IncA87-246(G144A) were performed using NAMD2.12 and the CHARMM36m protein forcefield. Bonds to hydrogen were constrained using the SHAKE and SETTLE algorithms for protein and solvent, respectively. Trajectory analysis was conducted with VMD45.
This work used the Extreme Science and Engineering Discovery Environment (XSEDE).

Nikon Element Software was used for image analysis.

For manuscripts utilizing custom algorithms or software that are central to the research but not yet described in published literature, software must be made available to editors/reviewers. We strongly encourage code deposition in a community repository (e.g. GitHub). See the Nature Research guidelines for submitting code & software for further information.

Data

Policy information about availability of data

All manuscripts must include a data availability statement. This statement should provide the following information, where applicable:

- Accession codes, unique identifiers, or web links for publicly available datasets
- A list of figures that have associated raw data
- A description of any restrictions on data availability

Raw data are available in the source data file for our manuscript entitled "Source Data- NCOMMS-18-37615A" and includes all the raw data used to generate the graphs presented in Figures 4c, 4d, 5b, and Supplementary Figure 11b. It also includes the original western blot used to generate Figure 4a, and supplementary figure 10.

Field-specific reporting

Please select the one below that is the best fit for your research. If you are not sure, read the appropriate sections before making your selection.

☒ Life sciences ☐ Behavioural & social sciences ☐ Ecological, evolutionary & environmental sciences

For a reference copy of the document with all sections, see nature.com/documents/nr-reporting-summary-flat.pdf

Life sciences study design

All studies must disclose on these points even when the disclosure is negative.

Sample size	No predetermine sample size calculation was performed. Functional assays were conducted with a minimum of 3 independent experiments with at least 100 cells/condition/experiments analyzed. Three independent experiments is the minimum number of samples for an accurate t-test analysis and the determination of statistical significance. We were able to detect statistically different means.
Data exclusions	No data were excluded.
Replication	All functional assays were verified by two different investigators. Furthermore, the various phenotypes were analyzed using different complementary approaches.
Randomization	There are no covariates impacting the functional assays. Cells were passaged no more than 10 times, ensuring that the cells did not mutagenize and derive from their original phenotype. Large stocks of each Chlamydia mutant were generated and aliquoted before use. For each experiment an aliquot from the original stock was used ensuring that each sample was identical.
Blinding	Samples were de-identified prior to analysis in order to remove any investigator bias.

Reporting for specific materials, systems and methods

We require information from authors about some types of materials, experimental systems and methods used in many studies. Here, indicate whether each material, system or method listed is relevant to your study. If you are not sure if a list item applies to your research, read the appropriate section before selecting a response.

Materials & experimental systems

n/a	Involved in the study
☐	☒ Antibodies
☐	☒ Eukaryotic cell lines
☒	☐ Palaeontology
☒	☐ Animals and other organisms
☒	☐ Human research participants
☒	☐ Clinical data

Methods

n/a	Involved in the study
☒	☐ ChIP-seq
☒	☐ Flow cytometry
☒	☐ MRI-based neuroimaging

Antibodies

Antibodies used	mouse anti-FLAG, clone M2 (Sigma, Lot# SLBR7936V, cat#F1804); goat anti-MOMP (Virostat, Lot# GF792, Cat#1621); donkey anti-mouse Alexa Fluor 555 (Invitrogen, Cat#A31570); donkey anti-goat Alexa Fluor 488 (Invitrogen, Cat#A11055)
Validation	All antibodies used are commercially available. From manufacturer web site: Anti-FLAG Specificity Conforms: Detects a single band of protein on a Western Blot from mammalian crude cell lysates by chemiluminescent probing. From manufacturer web site: Anti-MOMP antibody is specific to MOMP, it does not react with C. psittacii and C. pneumonia. Uninfected cells were negative.

An additional verification is performed in our lab based on the assessment of the number of bands obtained in western blot (usually only one), and the appropriate size of the band.

Eukaryotic cell lines

Policy information about cell lines

Cell line source(s)	HeLa cells were obtained from the American Type Culture Collection (ATCC) -CCL-2
Authentication	The cells used in this study were thawed from the original vials that were frozen as soon as the HeLa cells were purchased from ATCC, thus limiting any contamination with other cells.
Mycoplasma contamination	Cells are negative for mycoplasma contamination, and are tested every 6 months.
Commonly misidentified lines (See ICLAC register)	N/A
